# Deep Sequencing of Protease Inhibitor Resistant HIV Patient Isolates Reveals Patterns of Correlated Mutations in Gag and Protease

**DOI:** 10.1371/journal.pcbi.1004249

**Published:** 2015-04-20

**Authors:** William F. Flynn, Max W. Chang, Zhiqiang Tan, Glenn Oliveira, Jinyun Yuan, Jason F. Okulicz, Bruce E. Torbett, Ronald M. Levy

**Affiliations:** 1 Department of Physics and Astronomy, Rutgers University, Piscataway, New Jersey, United States of America; 2 Center for Biophysics and Computational Biology, Temple University, Philadelphia, Pennsylvania, United States of America; 3 Department of Molecular and Experimental Medicine, The Scripps Research Institute, La Jolla, California, United States of America; 4 Department of Statistics, Rutgers University, Piscataway, New Jersey, United States of America; 5 Infectious Disease Service, San Antonio Military Medical Center, San Antonio, Texas, United States of America; 6 Department of Chemistry, and Institute for Computational Molecular Science, Temple University, Philadelphia, Pennsylvania, United States of America; University of Minnesota, UNITED STATES

## Abstract

While the role of drug resistance mutations in HIV protease has been studied comprehensively, mutations in its substrate, Gag, have not been extensively cataloged. Using deep sequencing, we analyzed a unique collection of longitudinal viral samples from 93 patients who have been treated with therapies containing protease inhibitors (PIs). Due to the high sequence coverage within each sample, the frequencies of mutations at individual positions were calculated with high precision. We used this information to characterize the variability in the Gag polyprotein and its effects on PI-therapy outcomes. To examine covariation of mutations between two different sites using deep sequencing data, we developed an approach to estimate the tight bounds on the two-site bivariate probabilities in each viral sample, and the mutual information between pairs of positions based on all the bounds. Utilizing the new methodology we found that mutations in the matrix and p6 proteins contribute to continued therapy failure and have a major role in the network of strongly correlated mutations in the Gag polyprotein, as well as between Gag and protease. Although covariation is not direct evidence of structural propensities, we found the strongest correlations between residues on capsid and matrix of the same Gag protein were often due to structural proximity. This suggests that some of the strongest inter-protein Gag correlations are the result of structural proximity. Moreover, the strong covariation between residues in matrix and capsid at the N-terminus with p1 and p6 at the C-terminus is consistent with residue-residue contacts between these proteins at some point in the viral life cycle.

## Author Summary

Understanding the structure of HIV proteins and the function of drug-resistant mutations of these proteins is critical for the development of effective HIV treatments. Selected *gag* mutations have been shown to provide compensatory functions for protease resistance mutations and may directly contribute to the development of drug resistance. To determine associations between protease inhibitor mutations and *gag*, we utilized deep sequencing of HIV *gag* and protease from a collection of viral isolates from patients treated with highly active retroviral protease inhibitors. Deep sequencing allows for accurate measurement of mutation frequencies at each position, allowing estimation, using a novel method we developed, of the covariation between any two residues on *gag*. Using this information, we characterize the variation within *gag* and protease and identify the most strongly correlated pairs of inter- and intra-protein residues. Our results suggest that matrix and p1/p6 mutations form the core of a network of strongly correlated *gag* mutations and contribute to recurrent treatment failure. Extracting *gag* residue covariation information from the deep sequencing of patient viral samples may provide insight into structural aspects of the Gag polyprotein as well new areas for small molecule targeting to disrupt Gag function.

## Introduction

Despite great advances in the treatment of HIV/AIDS, the rapid evolution of resistance against protease inhibitors (PIs) contributes significantly to the persistence of highly active retroviral (ART) failure. Resistance mutations in the viral protease (PR) have been extensively studied [1–5], but mutations in its substrate, Gag, have been less well-studied and drug resistant mutations not as well cataloged. Protease inhibitor-mediated mutations in *gag* function as compensatory mutations for protease function and can directly promote resistance to PIs [6–14]. Investigation of resistance mutations in protease has led to advancements in protease inhibitor development. A better understanding of the association among inhibitor resistance mutations in Gag and their contribution to PI failure could be useful for the design of maturation inhibitors and clinical treatment strategies, and for building structural models.

During the past decade, advancements in DNA sequencing technologies have allowed for the study of the viral populations within an individual, and importantly these advancements allow for the quantification of low and infrequent HIV drug resistant mutations, which are difficult to detect using traditional Sanger sequencing [15–17]. Moreover, it has been reported that viral mutations that occur with frequencies less than 10% are systematically under-measured with conventional sequencing techniques [18,19]. Importantly, deep-sequencing technologies can reliably detect sequence variants with frequencies of 1% or less when template tagging such as PrimerID is utilized [20,21].

The sequencing depth afforded by deep-sequencing comes with a cost, as the templates being sequenced, typically 75–200bp in size, are often smaller than the region of interest, thus disrupting linkage analysis. Even when paired-end read methodology is used, it is nearly impossible to determine if two mutations far apart in a sequence occur simultaneously. These limitations have forced most studies to focus on analyzing the frequencies of single residue substitutions. Little progress has been made in identifying pairs or higher order patterns of residue substitutions in HIV samples from patients using deep-sequencing technologies. Additionally, due to the cost of deep-sequencing large regions of a target genome, comprehensive, simultaneous deep sequencing of viral samples from patients is not attempted on a regular basis.

An open question in better understanding protease inhibitor resistance is the role of *gag* mutations, both cleavage and non-cleavage site mutations, in contributing to resistance. To this end, we have relied on next generation sequencing of a 2 kb region encompassing the entire *gag* gene and the protease portion of the *pol* gene from 93 HIV positive patients undergoing ART which included a protease inhibitor. This patient population is unique in that all patients were followed after the first failure through the second treatment, of which approximately one-half of the patients failed treatment and the remaining patients controlled their virus [22]. Given our sequential patient sample collection, viral sample amplification methodology, and the precise sequence coverage from deep-sequencing, we calculated single-site residue frequency variation in *gag* and protease from the viral population from each patient sample. These studies allowed examination of the patterns of single amino acid substitutions in Gag and their correlations with repeated PI-therapy failure. Importantly, the comprehensive viral sample collection and sequencing methodology allowed us to investigate two central aspects of protease inhibitor resistance in protease and *gag*: single-site variation and two-site covariation.

Conventional analysis methods of two-site covariation, often conducted on multiple sequence alignments of full DNA sequences, are difficult to apply to our type of data set due to the limited read lengths provided by current deep-sequencing methodology and the presence of multiple viral species in each sample. To overcome this challenge, we have developed a statistical framework to estimate the probabilities of observing double mutants from the observed single-site marginal probabilities in each sample. This advantage over other methods allows for the aggregation of the probabilities from all samples into a single probability to which conventional covariation analyses can be applied. This then allowed us to utilize mutual information (MI) to calculate the pair correlations between pairs of positions in *gag*. The strongest of such correlations were identified and their implications for g*ag* structural propensities are discussed. The same statistical framework can be applied to other systems that have been sequenced with next-generation sequencing technologies.

## Results

### High concordance in SNP frequency between sequenced viral replicates from patients

A sequential collection of peripheral blood viral samples from patients undergoing ART, containing a protease inhibitor (PI) and combinations of nucleoside and non-nucleoside reverse transcriptase inhibitors (NRTIs and NNRTIs), provides a unique opportunity to evaluate mutational changes in the Gag polyprotein and protease over time as a function of protease inhibitor treatment. For patients from which samples were obtained, all ARTs contained a single PI, but included combinations of nucleoside and non-nucleoside reverse transcriptase inhibitors (NRTIs and NNRTIs). There are only two relevant PI therapies for each patient: the first of which all patients failed prior to sequencing (treatment with various regimens had failed to maintain long-term suppression of viral replication below the limit of detection (50–400 copies/mL, depending on the time of testing)), and a second therapy, in which a different prescribed PI was provided and patients were successfully treated and suppressed virus or continued to fail treatment. Of 93 patients entering treatment, 80 patients had definitive second therapy outcomes, defined as success (28 patients, viral levels below 100 copies/mL) or failure (52 patients treatment with various regimens had failed to maintain long-term suppression of viral replication below the limit of detection (100–400 copies/mL, depending on the time of testing)). For the purpose of sequencing, samples were considered for inclusion into the studies with >1000 copies/mL. If possible, additional samples were obtained from patients who failed the second therapy. The remaining 13 patients had varying levels of viral load slightly below 1000 copies/mL and samples were excluded given inadequate viral amounts for sequencing studies.

Current next generation sequencing technologies require a large amount of starting material that greatly exceeds the amount of viral RNA present in a typical clinical sample. Reverse transcription and amplification of the viral material by PCR could introduce bias based on stochastic resampling, leading to a final product pool that is not representative of the initial sample. To reduce the effect of resampling, we attempted to maximize cDNA production and usage by adopting a 1-step RT-PCR approach. This single round of PCR used 40 cycles and was sufficient to generate hundreds of nanograms of product for >95% of RNA samples. In contrast, some other studies [21,23] have relied on a nested PCR approach, which may contribute to resampling bias and increases the total number of PCR cycles.

**Fig 1:**
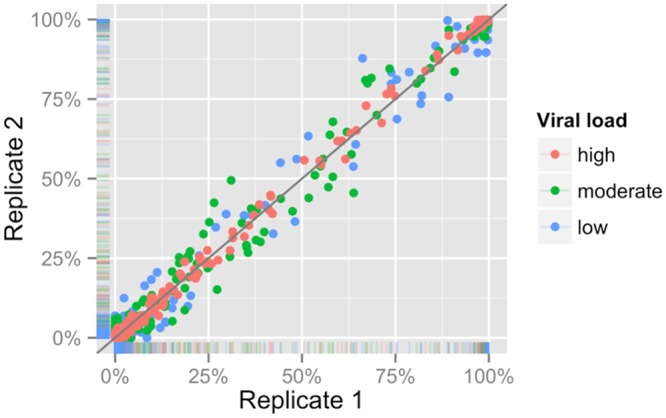
Single nucleotide polymorphism (SNP) frequencies between independent replicates are strongly correlated. Three patient samples with viral loads of 2,000, 8,500, and 67,000 copies/mL (low, moderate, and high, respectively), were extracted, reverse transcribed, amplified, and sequenced in duplicate. A comparison of SNP frequencies between these replicates shows R>0.99 in all cases. Even when ignoring SNPs that occur with <10% or >90% frequency in paired samples, R>0.95 for each pair.

To evaluate possible biases resulting from our RT-PCR procedure, we compared SNP frequencies in technical replicates, finding a high level of concordance. Specifically, for three clinical samples, we obtained multiple aliquots that were processed independently throughout the entire process of preparation and sequencing. These samples spanned a range of clinically relevant viral loads, from 2,000 copies/mL to 30,000 copies/mL. In each case, the paired replicates showed SNP frequencies that were well correlated even when ignoring SNPs that occur with <10% or >90% frequency in paired samples, R>0.95 for each pair (Fig 1). The difference between replicates appeared smallest for the sample with greatest viral load, indicating that a higher number of template molecules can reduce stochastic effects, as might be expected. Aside from the RT-PCR process, the high level of sequencing coverage afforded by the use of the Illumina HiSeq 1000 could also be a factor in the strong correlation between replicates.

The consensus among many studies using next generation sequencing is that discrimination of true variants from background variation becomes difficult at frequencies below 1% [16]. Additionally, recent work comparing sequencing with different primer tagging procedures shows that standard sequencing analysis, like has been conducted in this study, cannot distinguish true mutations from artificial mutations present at frequencies less than 1% [15]. However, at frequencies above 1%, standard sequencing analysis has a similar accuracy to that of primer tagged sequences. To ensure that mutations were likely to be biologically relevant we required that mutations occur with frequencies >1% (i.e., related to exposure to protease inhibitors) and occur in ≥5 different patient samples.

### Mutations in protease and *gag*

Mutations in protease that occur under protease inhibitor (PI) treatment have been documented extensively [1,3–7,24–26]. The mutational patterns in prior reports serve as a comparison standard to which we can compare the mutational patterns identified in protease using deep sequencing. Across all treatment samples, regardless of treatment, 50 drug-associated protease mutations at 33 different positions were identified. In each patient sample, these mutations tend to be either dominant or almost absent. Due to this bimodal nature of the mutations at each position within a sample, a mutation was labeled as present if the proportion of the mutation is greater than 1%, and as absent if the proportion of the mutation is less than 1%. By this categorization, we are able to compare the protease mutation pattern in each sample with patterns seen in bulk sequencing and reported in the Stanford HIV Drug Resistance Database [24,27] (shown in S1 Fig). Overall, the protease mutational patterns in our samples resemble patterns from the Stanford HIV Drug Resistance Database exposed to a single PI. This is consistent with the notion that our samples were obtained from patients treated with a minimum of 1 PI and a maximum of two PIs. In contrast, the Stanford Database also included sequences from more protease inhibitor experienced patients. The protease site-specific frequencies identified are shown in S2 Fig.

The majority of Gag mutations that have been associated with protease inhibitor resistance are confined to mutations within or near the 5 cleavage sites (CSs) separating the Gag proteins [6]. Currently, there are less than 65 mutations at 40 Gag positions reported that are PI- or maturation inhibitor-associated or found to covary with protease mutations [6]. Of the 65 PI-associated mutations, only 10 have been shown to directly contribute to PI-resistance; the remaining mutations have been observed only under PI-treatment, but are otherwise of unknown viral utility. Together, these mutations only represent residue variation of roughly 8% of *gag*, and half are located at cleavage sites, which include the 10 resistance mutations contributing to PI-resistance. Nevertheless, it has been noted using conventional sequencing that many polymorphisms exist in Gag [28].

**Fig 2:**
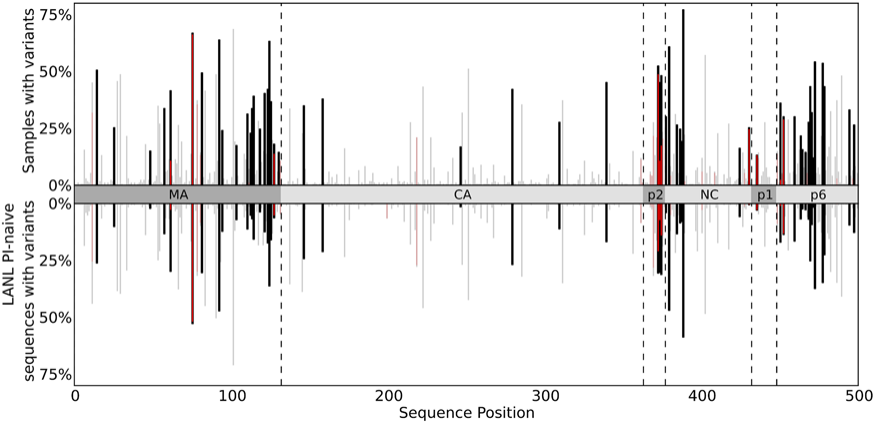
Samples with variants vs sequence position over Gag. Bar charts representing the number of samples in which amino acid variants are observed at each position in Gag derived from deep sequencing (top) and from 2378 drug-naive Gag sequences from LANL HIV sequence database (bottom). Variants shown from deep sequencing occur at frequencies above 1% in 5 or more patients and variants shown from LANL are present in at least 1% of sequences. The height of each vertical bar shows the number of patient samples with variants at Gag polyprotein positions. The different Gag proteins are indicated along the horizontal axis. Variants which have been documented in the literature as having associations with PI-exposure/resistance are shown in red. Positions at which the variation between the two datasets is small (|*f*_DS_-*f*_LANL_|<10%) are faded.

Utilizing deep sequencing, we found that residue variation in *gag* was abundant; shown in Table 1, we observe 329 residue changes at 192 positions throughout *gag*. In Fig 2, the observed variation in the deep sequencing data (top) is shown above with the variation present in 2378 drug-naive *gag* sequences from the Los Alamos HIV sequence database (bottom) (http://www.hiv.lanl.gov/). Positions in *gag* that have similar mutation frequencies between the two datasets are shown in light gray and with reported mutations linked to PI-exposure in red [6]. Mutations were identified within and just outside cleavage sites, but many mutations occurred outside the cleavage sites. We identified considerably more mutations from our deep sequencing data as compared with LANL data in the following regions of *gag*: in matrix both near the matrix/capsid (MA/CA) cleavage site and scattered throughout the central portion of MA; in p2 and nucleocapsid (NC) on either side of the p2/NC cleavage site; and throughout the first half of p6.

**Table 1:** Observed variation in each Gag protein.

Name		Length	Observed non-consensus amino acid mutations	Sites with at least one non-consensus amino acid mutation	
Gag		500	329	192	38%
	Matrix	132	122	64	48%
	Capsid	231	54	44	19%
	p2	14	22	9	64%
	Nucleocapsid	55	42	27	49%
	p1	16	12	7	44%
	p6	52	77	41	79%
Protease		99	75	46	46%

Our studies identified mutation positions currently associated with PI-resistance [6], as well as mutation variants associated with PI-exposure in cleavage sites. Cleavage site mutations that are associated with PI-resistance in the CA/p2 cleavage site, residues A360 and L363, and the NC/p1 cleavage site residue Q430, were not seen. In four of the five cleavage sites, defined as the region of 10 residues centered on the proteolytic site, mutations were identified that are not currently PI-associated (S1 Table). However, many of these mutations are variants of PI-associated mutations (S2 Table), and therefore could well be PI-associated. Of the observed cleavage site mutations that are known to be associated with PI resistance, excluding those in the highly variable p2/NC cleavage site, most occurred at relatively low frequency within each sample. However, NC/p1 and p1/p6 cleavage site mutations which we observe at low frequency have been shown to almost always appear in the presence of protease mutations that decrease inhibitor binding, such as D30, V82, I84, and M90 mutants [6].

HIV CA, and other selected domains of Gag, are necessary for assembly and are under pressure to maintain their functionality [6,29,30]. Regions of viral *gag* that are highly conserved, i.e., with low residue variability after drug treatment, are of interest given that these regions may be targets for future inhibitors. We examined the frequencies of mutated residues within the *gag* genes. The CA region demonstrated a mutated residue frequency accounting for 20% of its length, thus making it much more conserved than other Gag proteins (Table 1). A recent report has evaluated the viral fitness effects of single amino acid substitutions in CA [29]. Rihn et al. found that ~5% of all possible amino acid CA substitutions resulted in viruses that replicate *in vivo*. Moreover, engineered viruses were identified that contained “fit but rare” mutants that were <0.3% of or never found in 1000 HIV B patient sequences selected from the Los Alamos HIV database. The authors concluded that there exists some unknown selection pressure that selects against these particular high fitness mutations in individuals. However, an alternative possibility is that these high fitness mutants are present in viral populations from patients, but were too rare to be detected reliably in the authors’ reference sequences, which were obtained by conventional sequencing. To evaluate this possibility, we have examined whether 11 high fitness mutations presented in Rihn et al. (Table 9 of [29]), I2L, I6T, N21S, S33C, I91T, R100S, S149C, E187V, A204G, A209T, and A209V, were present in our dataset. Of these mutants, 9 were completely absent in the dataset. The remaining two mutants, N21S and I91T, occur infrequently. Thus, 9 of 11 fit but rare mutations identified by Rihn et al. were absent in both our dataset [29] and the Los Alamos HIV database.

### Mutations associated with therapy failure

**Fig 3:**
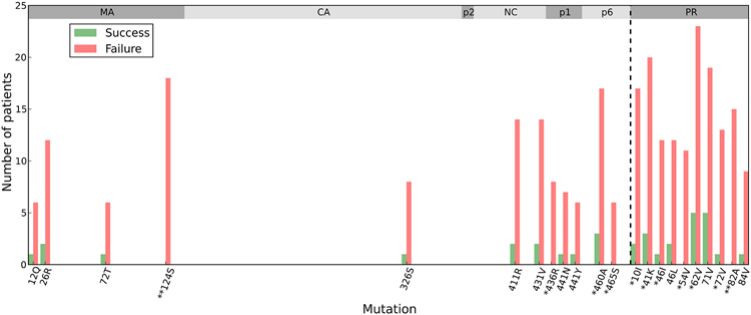
Mutations that are contribute to therapy failure. Bar charts showing the number of patients in which specific mutations occur during PI-based regimens including indinavir (IDV), saquinavir (SQV), and nelfinavir (NFV). Shown are the mutations with the largest differences between the successfully and unsuccessfully treated patient populations. Patients who failed therapy are shown in red and patients who had successful therapy are shown in green. The percentage above each bar denotes the percentage of all patients treated with that regimen which experience that mutation. Mutations with a */** are found to be statistically significant after Holm-Bonferroni correction with family-wise error rates 0.1 and 0.01 respectively among 140 mutations.

Next, we evaluated the differences in mutation variation in *gag-protease* between the viral samples obtained from patients that failed or were successful on ART. At the 599 *gag-protease* positions, 140 individual mutations are observed above 1% frequency in 5 or more patients who failed subsequent ART (which constitutes 10% or more of patients who failed therapy). From these 140 mutations, we identified 11 mutations that are significantly associated with repeated therapy failure when adjusted for multiple hypothesis testing: MA N124S, NC/p1 cleavage site K436R, p6 E460A and F465S, and PR L10I, R41K, M46I, I54V, I62V, I72V, V82A. The number of patients from each group in which these mutations were observed is shown in Fig 3. All of the protease mutations, except R41K, are associated with PI-resistance, including the NC/p1 cleavage site mutation K436R. The protease mutations M46I, I54V, and V82A have been shown to have a major impact on PI susceptibility, while L10I, R41K, I62V, I72V are accessory or polymorphic mutations [1,4,5,27]. The MA N124S mutation and two p6 E460A, F465S mutations have not been characterized as to their role in fitness or drug resistance, although N124 is proximal to the MA/CA cleavage site and could possibly function to enhance cleavage. Of the 22 mutations identified in Fig 3, which represents those with largest frequency differences between the viral sequences identified in failed and successfully treated patients, almost 90% of the total occur in MA, p1/p6, and protease. This finding closely parallels the observation shown in Fig 2, in which the majority of inhibitor-mediated mutational variation occurs in MA and p6.

### Covariation of mutations in Gag-protease proteins

Identifying pairs of correlated mutations from deep sequencing data is not as straightforward as when given conventional multiple sequence alignments. This problem arises given the short reads from deep sequencing templates, which results in loss of direct sequence linkage between distal genes, such as between *gag* and protease, and the presence of many viruses (distinct *gag* and protease combinations) in a population. Thus, determining the joint probabilities for observing changes in pairs of distal mutations cannot be readily determined. Furthermore, it is not clear how to aggregate these joint probabilities from the many sequencing reads obtained from a viral population. To provide an estimate of distal mutation variant frequency, we have designed a procedure with two main steps: In the first step, for any pair of residue positions, say one in *gag* and one in protease, we calculate bounds on the joint probability of observing a double mutant at that pair of residue positions from the marginal mutation probabilities obtained from each sample. We then estimate the joint probability from those bounds and then aggregate these joint probability estimates from each viral sample into a single estimate for the probability to observe that pair of mutations across all samples. In the second step, using these joint probabilities, we assess the correlation implicit in the joint probabilities involving two positions in the viral genome using mutual information (MI). We briefly explain the procedure below, for a more detailed description, see Materials and Methods.

For any pair of positions, we know with high-precision how likely each mutation is to occur independently of other mutations due to the extremely deep sequence coverage in each sample. These single-site probabilities constrain the possible values of the joint probability of the mutations occurring simultaneously. Within each viral sample, we find that the bounds on the double mutant probability are often very narrow because of the bimodal nature of the mutations, which are either dominant or almost absent at a typical position within a sample. Using estimates for the double mutant probability from each sample based on the bounding procedure described in Materials and Methods, we average the probabilities over all patient viral samples to get an estimate for the double mutant probability in the patient population. Although the number of samples is limited, because we are averaging probabilities and not a single binary count from each sample, this procedure produces a probability table for the joint probabilities for the status of two mutants that differs from how a table of counts is directly constructed from a multiple sequence alignment. As a result, given tight bounds, the joint probability estimates are much more precise than those calculated from table counts. In our study, for each pair of positions, we construct a 2×2 probability table representing the probabilities of observing both wildtype residues, a single amino acid substitution at the first position, a single amino acid substitution at the second position, and amino acid substitutions at both positions.

Utilizing this methodology we can identify co-evolving pairs of mutations by searching for pairs of mutants with average double mutant probabilities that differ greatly from independence. We quantify this deviation by the mutual information (MI); pairs with the largest mutual information have the strongest covariation. The details of this procedure are explained in Materials and Methods.

### Validation of bivariate marginal estimation

It is crucial for our method to be validated on a dataset where correlations between two positions are known. A simple test dataset can be constructed from the deep sequencing pileup from each sample in which pairs of PR residues are close enough in sequence to be on the same set of 100bp paired end reads. Doing so provides us with a test dataset which mirrors the structure of our true dataset, but the bivariate marginal distributions are known. From the known bivariate marginals, we can compute the univariate marginal distributions from which we then estimate bounds on the bivariate marginals via our bounding procedure.

**Fig 4:**
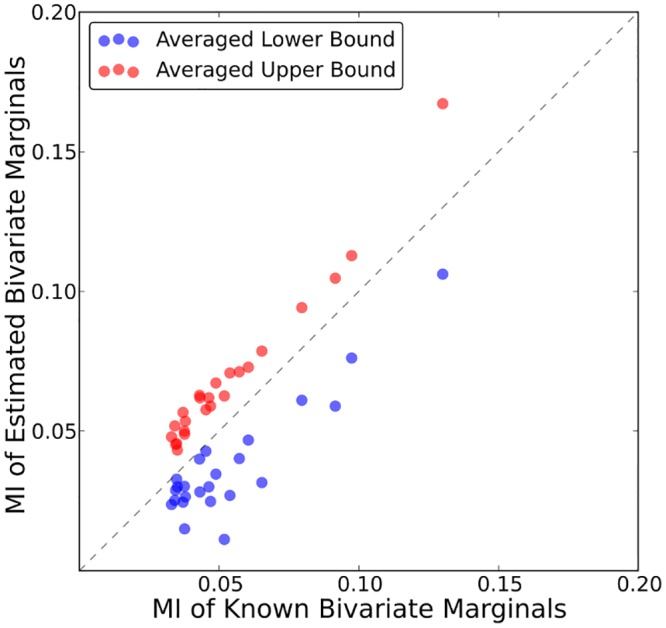
MI of estimated bivariate marginals versus MI of known bivariate marginals. Shown is the MI for 24 PR-PR pairs computed using the estimated bounds on the bivariate marginals (lower bound—red, upper bound—blue) versus using the known bivariate marginals.

We have calculated bivariate counts from the deep sequencing pileup for 24 pairs of PR residues which have previously been shown to be correlated using conventional sequencing [3,4] where we had sufficient pileup (>10,000 overlapping reads). The estimated bounds (shown in S5 Fig) are typically very narrow, and the lower bound is often a very conservative estimate of the double mutant probability in each sample for all pairs examined. The mutual information (MI) computed for each pair using the bounding procedure is in good agreement with the MI computed using the known bivariate marginal probabilities, as shown in Fig 4. In practice, extracting the pileup can be computationally expensive for large regions of interest where the coverage is very high, and for systems with short reads, this method only allows for covariation analysis for residues that are relatively close together. Using our bounding procedure provides a computationally fast method to estimate bivariate marginals from single-site frequency counts for all pairs of interest, not just those within 200–300bp.

### Correlation analysis in using bound estimates protease captures known pair correlations

Protease mutation covariation in PI resistant viruses has been extensively studied and reported by Shafer and others [3–5]. Findings in these publications potentially serve as a benchmark that can be used to estimate how well we are able to recover information about correlated mutations from protease and *gag* deep sequencing data using the bounding procedure. Using a multiple sequence alignment (MSA) of 4919 treated protease sequences from the Stanford HIV Database (HIVDB, [24]); we computed the MI for all 4851 (99 choose 2) pairs of positions. Among the 1594 pairs with positive, nonzero MI extracted from the MSA, 1275 pairs are common to our deep sequencing dataset; we chose these 1275 PR-PR positively correlated pairs to assess our bounding procedure. From these 1275 pairs, the 127 (top 10%) pairs with the highest MI when calculated using the MSA were selected as the putative true positives to which we compared our procedure. These 127 pairs closely resemble the pairs of positions [4,5] and corresponding pairs of mutations [3] identified as highly correlated in previous work [3–5].

**Fig 5:**
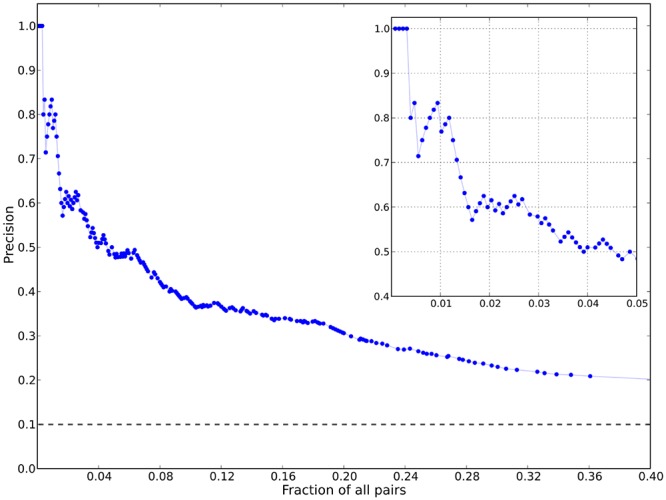
Recovery of correlated protease-protease pairs. Shown is a plot of the precision (also known as positive predictive value (PPV)) for the top 40% of correlated PR-PR pairs ranked by mutual information. In total, 1275 pairs are plotted (127 putative true positives) that are common to both our deep sequencing dataset and the Stanford HIVDB downloadable protease dataset (see Materials and Methods). True positives were determined through a mutual information calculation similar to the calculations in [3]. The precision plot shows, given a value of MI^0^, the number of true positives with MI>MI^0^ identified with deep sequencing divided by the number of true and false positives with MI>MI^0^ versus the percentage of all pairs with MI>MI^0^. The dashed horizontal line indicates the PPV of randomly drawn pairs. The insert shows the PPV for the top 5% of PR-PR correlated pairs ranked by MI.

Fig 5 shows the recovery of the 127 pairs with highest MI from the Stanford Database in our dataset. In the most strongly correlated pairs, we recover several well-studied strongly correlated pairs of protease mutations, such as D30-N88, I54-V82, and E35-M36. The 20 most strongly correlated pairs are shown in Table 2. We observe an 8-fold enrichment within the top 1% of deep sequencing pairs with highest MI (Fig 5, insert), and 5-fold enrichment within the top 5% of deep sequencing pairs with highest MI (S3 Table). The recovery is uniformly higher if the least conservative bound on the double mutant probability is used, and a comparison is shown in S3 Fig. But it is evident that below the pairs with the largest MI values, which are consistent between the two databases, there are many pairs identified in the deep sequencing dataset as correlated that are not identified in the Stanford HIVDB. These differences are not likely due to sample size effects in the relatively small number of patient samples in this study because the univariate marginals and the bivariate marginal estimates are calculated with high precision in each sample due to the extremely high coverage afforded by deep sequencing and the very narrow bounds imposed on the bivariate probabilities by the univariate probabilities. We believe this discrepancy is due to real differences between the two sets of data, specifically that joint probabilities are systematically under-estimated using conventional sequencing technologies. It has been shown that variants with frequencies less than 35% are difficult to detect with conventional Sanger sequencing, and protease and *gag* mutations with frequencies less than 10% often go undetected using standard genotyping analysis [18,19]. Yet with deep sequencing, even without template tagging, we are able to reliably detect variants with frequencies as low as 1% in each patient sample. For example, we observe positions L24-T74 to be strongly correlated in the deep-sequencing dataset (5th highest PR-PR pair by MI), but this pair is not found to be correlated using the HIVDB MSA. We have confidence this correlation exists because individual mutations L24I and T74S/P/A have strong associations with PIs [4,26]. The double mutant probability for this pair is estimated between 2.18–2.19% in the deep sequencing dataset but is 0.91% in the HIVDB. With reliable detection of low frequency mutants, deep sequencing allows us to more accurately identify correlations between pairs of residues that are difficult to detect with conventional sequencing.

**Table 2:** Top 20 most strongly correlated pairs of PR-PR positions.

Protease Position 1	Protease Position 2	Observed in HIVDB^a^	Position 1 PI-association^b^	Position 2 PI-association^b^	MI	P_xy_	P_xy_^0^	P_x_	P_y_
30	88	Yes	Yes	Yes	0.128	0.056	0.006	0.072	0.082
54	82	Yes	Yes	Yes	0.088	0.072	0.018	0.088	0.200
73	90	Yes	Yes	Yes	0.072	0.055	0.013	0.064	0.201
46	82	Yes	Yes	Yes	0.070	0.096	0.034	0.170	0.200
24	74	No	Yes	Yes	0.061	0.022	0.001	0.027	0.045
35	36	Yes	Yes	Yes	0.053	0.096	0.040	0.204	0.195
69	84	No	No	Yes	0.049	0.023	0.002	0.040	0.050
24	46	Yes	Yes	Yes	0.046	0.026	0.005	0.027	0.170
24	82	Yes	Yes	Yes	0.042	0.027	0.005	0.027	0.200
13	33	Yes	Yes	Yes	0.042	0.034	0.008	0.174	0.043
10	93	Yes	Yes	Yes	0.042	0.156	0.094	0.282	0.334
12	19	Yes	No	No	0.041	0.031	0.005	0.088	0.061
33	66	No	Yes	Yes	0.040	0.017	0.001	0.043	0.026
10	46	Yes	Yes	Yes	0.040	0.098	0.048	0.282	0.170
32	82	Yes	Yes	Yes	0.040	0.027	0.006	0.028	0.200
24	64	No	Yes	No	0.039	0.024	0.004	0.027	0.140
37	63	No	No	Yes	0.038	0.284	0.233	0.309	0.752
33	60	No	Yes	Yes	0.038	0.027	0.004	0.043	0.101
41	93	No	No	Yes	0.038	0.143	0.085	0.256	0.334
30	35	No	Yes	Yes	0.036	0.046	0.015	0.072	0.204

^a^Residue pair is among the top 10% most strongly correlated pairs in the Stanford HIVDB as ranked by mutual information.

^b^PI-association of protease positions determined in literature [1,4,6].

As additional evidence that we observe meaningful correlations derived from the deep sequencing using our bounding procedure to constrain the bivariate probabilities, we note that many of the apparent false positive pairs of mutations in protease identified in our analysis may be biologically important because these pairs contain at least one variant associated with PI-exposure. For example, mutations at pairs of positions such as D30-E35, E35-V84, and M36-N88 have been shown individually to directly reduce drug susceptibility. D30 and V84 are located in the protease active site and thus these pairs of positions suggest we observe compensatory-active site pairs previously under sampled due to the limitations of conventional sequencing techniques. Moreover, in the top 5% of pairs with highest MI from deep sequencing, 34 of the 58 pairs identified as putative false positives involve at least one known resistance mutation. The fact that many of the putative false positives detected via our procedure are combinations of PI-associated residues suggests that the true recovery of strongly covarying pairs of protease positions using our bounding procedure is likely significantly higher than the apparent recovery rate shown in Fig 5.

### Strongest correlations in Gag indicate functional and structural patterns

We turn now to a consideration of correlations within the Gag polyprotein and between Gag and protease. Tables 3 and 4 show the strongest 20 positively correlated pairs for Gag-PR and Gag-Gag; the top 1% positively correlated pairs with highest MI values for each region are displayed in S4 and S5 Tables respectively.

**Table 3:** Top 20 most strongly correlated pairs of Gag-PR positions.

Gag Position	PR Position	Gag Protein	Protease PI-association^b^	MI	P_xy_	P_xy_^0^	P_x_	P_y_
431	82	NC/p1 CS	Yes	0.085	0.098	0.032	0.158	0.200
431	46	NC/p1 CS	Yes	0.071	0.085	0.027	0.158	0.170
431	10	NC/p1 CS	Yes	0.063	0.106	0.045	0.158	0.282
8	57	MA	No	0.058	0.023	0.002	0.024	0.086
264	76	CA	Yes	0.056	0.012	0.000	0.013	0.014
486	37	p6	No	0.055	0.076	0.029	0.094	0.309
159	37	CA	No	0.053	0.163	0.093	0.300	0.309
443	35	p1	Yes	0.048	0.030	0.006	0.030	0.204
465	76	p6	Yes	0.046	0.012	0.000	0.020	0.014
375	37	p2/NC CS	No	0.046	0.163	0.097	0.313	0.309
119	37	MA	No	0.045	0.070	0.029	0.092	0.309
326	57	CA	No	0.044	0.038	0.008	0.088	0.086
65	43	MA	Yes	0.044	0.021	0.002	0.058	0.030
163	72	CA	Yes	0.044	0.037	0.008	0.052	0.157
453	36	p1/p6 CS	Yes	0.044	0.075	0.029	0.149	0.195
182	16	CA	No	0.044	0.018	0.001	0.050	0.023
486	24	p6	Yes	0.043	0.022	0.003	0.094	0.027
456	24	p6	Yes	0.043	0.027	0.005	0.194	0.027
410	20	NC	Yes	0.042	0.018	0.002	0.018	0.087
431	93	NC/p1 CS	Yes	0.042	0.104	0.053	0.158	0.334

^a^PI-association of protease positions determined in literature [1,4,6].

**Table 4:** Top 20 most strongly correlated pairs of Gag-Gag positions.

Gag Position 1	Gag Position 2	Gag Protein 1	Gag Protein 2	MI	P_xy_	P_xy_^0^	P_x_	P_y_
228	248	CA	CA	0.214	0.160	0.048	0.168	0.288
159	280	CA	CA	0.144	0.219	0.102	0.300	0.341
46	75	MA	MA	0.108	0.069	0.012	0.130	0.094
123	443	MA	p1	0.098	0.030	0.002	0.050	0.030
163	443	CA	p1	0.097	0.030	0.002	0.052	0.030
12	46	MA	MA	0.095	0.121	0.052	0.398	0.130
348	443	CA	p1	0.093	0.030	0.002	0.058	0.030
163	418	CA	NC	0.086	0.049	0.009	0.052	0.168
63	66	MA	MA	0.086	0.024	0.001	0.025	0.037
182	186	CA	CA	0.077	0.036	0.004	0.050	0.071
173	342	CA	CA	0.076	0.050	0.009	0.142	0.064
75	443	MA	p1	0.074	0.030	0.003	0.094	0.030
119	443	MA	p1	0.074	0.030	0.003	0.092	0.030
403	418	NC	NC	0.073	0.151	0.083	0.498	0.168
242	248	CA	CA	0.072	0.091	0.033	0.116	0.288
397	404	NC	NC	0.072	0.023	0.001	0.043	0.025
387	398	NC	NC	0.072	0.042	0.006	0.094	0.061
123	348	MA	CA	0.071	0.032	0.003	0.050	0.058
340	495	CA	p6	0.067	0.111	0.048	0.317	0.151
79	81	MA	MA	0.066	0.075	0.025	0.255	0.097

For the Gag-PR pairs shown in Table 3, the NC/p1 cleavage site residue A431 is well represented and strongly correlated with protease residues associated with major PI-resistance V82, M46, L10, and L93. Association between A431V and protease mutations V82A and M46L has been demonstrated using *in vitro* mutagenesis experiments [31] and regression analysis [14], but the correlations between A431 and both L10 and L93 have not been previously reported. Additionally, mutations at the p1/p6 CS, P453 and L449, are strongly correlated with PI-associated protease positions M36 and I84 respectively. L449-I84 has been previously observed [14], but P453-M36 is not mentioned in the literature. It has been recently reported that mutations in the p1/p6 cleavage site are correlated with PR resistance mutations D30N and N88D [32], and while we observe no pairs in the top 1% that involve these PR mutations, within the top 5%, R452/P453-D30 and R452/P453-N88 are present. There is no previous evidence of mutations at the CA/p2 CS that are associated with PR mutations in the literature, and while we find positions in 4 of the 5 Gag CSs that are strongly correlated with positions in PR, we also find no evidence that positions at the CA/p2 CS are strongly correlated with positions in PR.

Outside of the CSs we find that the overwhelming majority of Gag positions strongly correlated with mutations in protease are located within the first 200 residues (MA/CA) or the last 60 residues (p1/p6) of Gag. Although some positions in PR only appear correlated with positions in specific Gag proteins, such as A118/V128-I66 and T456/L486-L24, the majority of positions in PR shown in Tables 3, S4 (E35, N37, R41, and L93) are correlated with residues on opposite sides of the Gag polyprotein.

For Gag-Gag pairs, many intra- and inter-protein pairs are represented. The majority of the pairs in Table 3 that involve cleavage site residues appear in the NC/p2 cleavage site, which has been shown to be PI-sensitive and highly variable in bulk sequencing. Additionally, 20% of the 70 pairs shown in Table 3 involve the p1 residue G443, which is located near the *gag-pol* frameshift-regulating region and is flanked by several positions associated with PI-exposure mutations, K436, I437, and L449 [33].

Although there is little analysis of covariation of non-CS Gag mutations currently in the literature, residues R76, Y79, and T81 have been described as co-evolving under drug pressure [34]. These residues are all located on an alpha helix in the folded MA protein and it is theorized that mutations in this alpha helix allow greater flexibility in the secondary structure, which may enhance MA/CA cleavage site accessibility to the protease. We also observe strong correlations between residues in this region of MA as pairs Y79-T81 and V82-T84 are highly correlated (Tables 4, S5). Additionally, MA residue L75 is highly correlated with several residues in other Gag proteins.

While Gag is not the primary target of protease inhibitors, we observe the correlations as measured by mutual information within Gag proteins are of similar magnitudes as in protease (Tables 2, 3, 4). The most strongly correlated pair of positions identified is between two CA residues, which as can be observed from their crystal structures are in close proximity (PDB 3MGE, 2M8L, 3P05, 3MGE). This is also observed for several other strongly correlated pairs of residues on the same Gag protein (see Materials and Methods for a full list of PDB IDs). Table 5 shows the heavy atom-atom distances between wild type residues for the strongest 20 intra-Gag protein and-protease correlations for which structures exist in the PDB file (atom-atom distances for all intra-protein pairs from Tables 2, 3, 4 are listed in S6 Table). We observe 11 of these 20 positions to be within 8Å within the mature protease and Gag proteins. It is apparent that ranking pairs of residues by MI provides major enrichment for detecting structural proximity over random sampling; for example, measuring the distance between 20 randomly chosen pairs from the CA pentamer dimer will yield 11 pairs in close proximity with probability less than 10^-14^. All available multimerizations of these proteins were examined for structural contacts: MA (monomer, trimer), CA (monomer, dimer, pentamer, hexamer), NC (monomer), PR (dimer); see Materials and Methods for more details. We find that the pair of positions with the largest mutual information identified in this study, Gag M228-G248, is within 6Å in all CA structures. The second most-strongly correlated pair, Gag V159-T280, is close in some CA dimer structures (<7Å) in the NMR structure. These residues may be functionally important in the multimerization of CA as they are on α-helices that play integral roles in dimerization and the formation of the hexameric CA lattice [35].

**Table 5:** All-(heavy)-atom distances between top 20 most strongly correlated intra-protein as identified by mutual information.

Protein	Pos 1	Pos 2	Res 1	Res 2	MI	Smallest R_ij_ (Å)	Structure^a^	PDB Model^b^	Chains	Atoms
CA	228	248	MET	GLY	0.21	**3.1**	CA2	22	BB	CE-O
CA	159	280	VAL	THR	0.14	**6.4**	CA2	39	AA	O-OG1
PR	30	88	ASP	ASN	0.13	**3.7**	PR2	0	AA	CA-OD1
MA	46	75	VAL	LEU	0.11	**6.7**	MA3	0	AC	CA-CD2
MA	12	46	GLU	VAL	0.10	**16.6**	MA	11	AA	OE1-CG1
PR	54	82	ILE	VAL	0.09	**8.2**	PR2	0	BB	CD1-O
MA	63	66	GLN	PRO	0.09	**3.0**	MA	14	AA	O-CG
CA	182	186	GLN	THR	0.08	**2.4**	CA2	10	AA	OE1-OG1
CA	173	342	SER	THR	0.08	**14.3**	CA2	69	AB	OG-O
NC	403	418	GLY	ASN	0.07	**5.5**	NC	9	AA	CA-ND2
CA	242	248	THR	GLY	0.07	**7.6**	CA5	0	EE	O-CA
NC	397	404	LYS	LYS	0.07	**6.6**	NC	5	AA	O-O
NC	387	398	THR	GLY	0.07	**13.8**	NC	9	AA	O-O
PR	73	90	GLY	LEU	0.07	**10.6**	PR2	0	BB	CA-CA
PR	46	82	MET	VAL	0.07	**14.6**	PR2	0	BA	O-CG2
MA	79	81	TYR	THR	0.07	**4.0**	MA	19	AA	O-CA
NC	390	401	ASN	LYS	0.07	**16.1**	NC	5	AA	ND2-NZ
CA	173	248	SER	GLY	0.07	**20.4**	CA5	0	BB	O-O
MA	46	119	VAL	ALA	0.06	**17.7**	MA	14	AA	CG2-CB
CA	146	148	ALA	SER	0.06	**3.9**	CA5	0	DD	O-CA

Listed are the smallest atom-atom distances (only heavy atoms, excluding side chains) for the most strongly correlated pairs of residues from the three regions PR-PR, Gag-PR, Gag-Gag ranked by MI in representative structures. For structures with multiple chains, inter-chain distances were computed and the chain and atom combinations of the smallest calculated distance for each pair are listed.

^a^MA: matrix monomer, PDB 2H3F; MA3: matrix trimer, PDB 1HIW; CA: capsid monomer, PDB 3MGE; CA2: capsid dimer, PDB 2M8L; CA5: capsid pentamer, PDB 3P05; CA6: capsid hexamer, PDB 3MGE; NC: nucleocapsid monomer, PDB 2EXF; PR2: protease dimer, PDB 1ODW.

^b^For PDB files with multiple structural models, the model number with the smallest atom-atom distance is listed.

**Fig 6:**
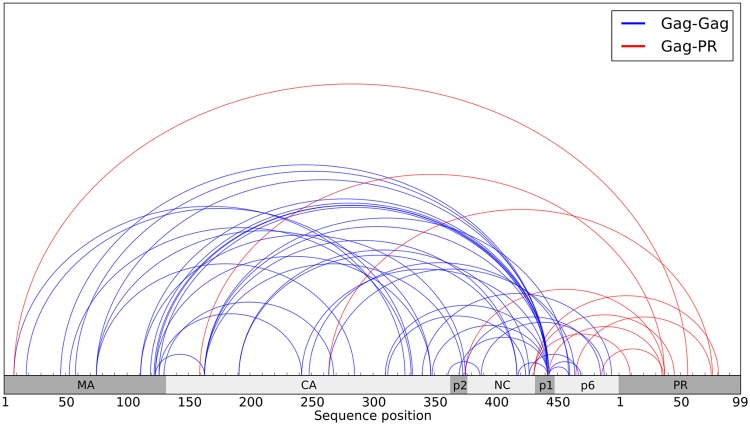
Strongest inter-protein correlations among PR and Gag proteins. Each semicircle denotes a correlation between the positions located at its endpoints. The strongest 50 correlations as measured by mutual information (MI) from the following regions are shown: Gag-Gag (blue), Gag-PR (red).

We also examined the distribution of inter-protein correlations among Gag proteins and protease and we observe that more than 300 residues separate some of the strongest Gag inter-protein correlations in sequence, shown in Fig 6. There are no complete crystallographic or NMR structures containing two or more of the large Gag proteins. However, models have been constructed to simulate the structural propensities of a mutated Gag intermediate comprised of MA, CA, p2, and NC, all of which involve the MA domain folding over the CA domain, and it is theorized these structures occur due to entropic effects disfavoring straight conformations of the polyprotein [36]. We observed strong MA-CA correlations that are consistent with these models, and moreover, we find many strong correlations between the MA/CA proteins and residues in p1/p6. One explanation for these very long-range Gag correlations could be transient structural contacts between the p1/p6 proteins and the MA/CA fold in the immature Gag polyprotein at some point in the viral life cycle. A possible model for the full polyprotein is a distorted circular structure; with a fold near either side of p2, the C-terminus proteins could interact directly with the MA/CA hairpin structure previously theorized [36]. However, further analysis is needed to distinguish correlations arising from direct spatial proximity from those that are due to networks of indirect effects [37].

## Discussion

To the best of our knowledge, no previous study has attempted to use multiple deep sequencing samples from PI resistant patients as a population from which to infer correlated mutation pairs. Typically, this type of analysis has been done by examining the one- and two-site amino acid frequency counts at each position in multiple sequence alignments. Previous to this study, there was no direct method to extract two-site frequency counts from viral deep sequencing data of *gag* and *pol* given the absence of sequence linkage due to the short sequencing reads. In order to identify strongly correlated residues through the entire 2 kb region of *gag* and *pol* we sequenced, we developed a procedure that estimates the bivariate joint probabilities from the observed single-site frequencies. This is made possible by the high precision with which the single-site frequencies are calculated in each sample due to high coverage, and by the bimodal nature of the single-site frequencies in each sample, which yield tight bounds on the bivariate probabilities. Although we considered the simplest such procedure, which involves estimating bounds on the four pair probabilities (M, M), (M, W), (W, M), and (W, W), where W denotes the consensus amino acid and M denotes any amino acid substitution, similar to [4,38], this procedure can be expanded to consider pairs of all individual amino acid substitutions instead of grouping all substitutions together. Recent advances in sequencing and library construction may allow the two-site frequencies to be observed directly from longer sequencing reads [21,39], but until this methodology becomes more widely available, our procedure provides a way to extract meaningful two-site frequencies from short, non-physically linked reads.

However, our procedure is reliant on the ability to distinguish the population within individuals from the entire population consisting of all the individual samples. This particular concern is often absent in the analysis of collections of sequences obtained from traditional sequencing where typically only one sequence is sampled from one individual, and the only analysis to be done is to examine the patterns of sequence variation across individuals. The use of deep sequencing expands upon this by allowing the variation within individuals to be studied. When there is considerable variation within, it becomes difficult to untangle the variation across patients from that present within individuals. As discussed previously, the frequency of mutations within each sample is typically limited to a bimodal present-or-absent pattern, which allows for analysis of covariation across samples.

The covariation analysis performed in this study relies on the frequency counts measured from the viral sequence population within each patient. The viral population within each patient has descended from founder viruses and the population at the time of sampling may have some background correlation due to phylogenetic similarity. Covariation analysis can be confounded by such phylogenetic effects [40–44], and a large literature has developed to account for such biases [44,45]. Although we have not accounted for this bias in our analyses, there are a number of factors that would argue against the covariation uncovered in our analyses being the result of simple inheritance from founder viruses. Firstly, strong selection pressure can create the environment for convergent evolution in which covariation dominates over phylogenetic effects [42,46,47]; indeed, drug resistance selection from reverse transcriptase (RT) inhibitors has been reported to generate a higher evolution rate in RT, thus fixing mutations, as compared to viral genes not under not under drug selection, such as envelope [48]. Secondly, in our covariation analysis, we have considered the potential influence of phylogeny across samples, evaluated the effects on MI, and found the effects to be minimal (see Materials and Methods).

In the patient samples used in this study, the protease and gag population diversity within an individual sample is typically limited. As a result the bounds on the bivariate probabilities are very tight and therefore we are able to aggregate the joint probabilities from the individual samples to extract meaningful information about population covariation. The fact that we examine a portion of the HIV genome which is relatively conserved, when compared, for example, to HIV *env* gene proteins [49] under immune selective pressure, influences the effectiveness of our analysis. Proteins or protein families evolving very rapidly under genetic drift and other forms of natural variation may not necessarily satisfy these conditions. Nevertheless, the procedure we have developed for identifying covariation from deep sequencing data with short reads used on single site mutation frequencies and bounds on joint marginals serves as a good starting point upon which future studies may expand datasets containing many deep sequenced samples.

As inhibitor potency increases, mutation pathways which confer resistance become more complex and involve more amino acid substitutions to compensate for major resistance mutations. Very few PI-associated mutations and even fewer resistance mutations have been previously identified outside of the protease and Gag cleavage sites [6]. By examining the patterns of amino acid substitutions in HIV Gag, we find evidence for new patterns of resistance. Mutations in the MA, and p6 proteins are much more prevalent in PI-experienced individuals who have failed therapy versus individuals who had successful therapy. All patients failed the initial ART, which include PIs, and thus most patients were found to have major PI-resistance mutations in protease. Gag mutations are likely accessory mutations, but the mechanisms by which most Gag mutations compensate for major resistance mutations are not known [50,51]. Recently Breuer et al. has proposed that PI-mediated cleavage and non-cleavage site mutations function to enhance the catalytic efficacy of PI-resistance proteases [52]. Mutations identified in this study that contribute to repeated therapy failure may “prime” the viral populations for major resistance mutations in subsequent therapies by enhancing catalytic efficacy of PI-resistant proteases, pre-compensating for resistance mutations with high fitness costs.

The majority of the Gag-protease correlations we observe have not been previously studied and these findings serve as a basis for future research into the resistance mechanisms in these regions. We observe positions in MA, CA, p1, and p6 which are strongly correlated with positions in protease at which major resistance mutations occur. This suggests that residues outside of the cleavage sites can influence protease function, and furthermore, resistance mechanisms in HIV are mediated not just by a few major resistance mutations, but a larger network of residues spanning the Gag polyprotein. It has been shown that the cleavage of Gag proteins is highly dependent on the sequence of the entire cleavage site and possibly proximal residues as well [52,53]. Similarly, correlated networks of amino acid mutations across Gag proteins likely contribute more strongly to the development of resistance than single amino acid substitutions.

Recent studies have examined covariation among Gag residues in drug naïve patients as the result of immune-pressures [54–58], but in the context of PI-exposure, very little has been published concerning the covariation of Gag residues with other Gag residues. Despite lacking directly accessible two-site frequencies, we are able to identify several strong signals of covariation in Gag. The magnitudes of the correlations we observe among positions in the Gag polyprotein are as large as those observed in protease. Although covariation is not direct evidence of structural propensities, this information can be useful for solving the structure of the Gag polyprotein at low resolution. We identify strong covariation between residues in MA, CA, p1, and p6 proteins, which suggests that p1 and p6 may be proximal to MA and CA regions of the Gag polyprotein at some point in the viral life cycle. The Gag polyprotein is believed to multimerize via first dimerizing in the CA domain [35]; the model suggested by the pattern of MI values is consistent with this thinking as it leaves CA exposed. There exist several methods, some based on mutual information, which have been developed to extract direct structural contacts (typically <8Å) from multiple sequence alignments [37,59–63]; it is possible to adapt these methods to detect direct structural propensities using covariation extracted from deep sequencing. In fact, due to the limited number of publicly available, full length Gag sequences, the bivariate probabilities estimated here may be better suited to parameterize these models than two-site frequency counts extracted from an MSA. When information about correlated mutations is combined with lower resolution structural information from experiments to determine structural contacts, like small angle X-ray scattering (SAXS) or hydrogen-deuterium exchange, more consistent models can be constructed for the structural ensembles that represent the Gag polyprotein which are needed for the interpretation of functional studies.

## Materials and Methods

### Deep sequencing data

The serum/plasma patient specimens were obtained from the U.S. Military HIV Natural History Study (IDCRP-000-03) as part of the Infectious Disease Clinical Research Program (IDCRP). All samples received by The Scripps Research Institute from the U.S. Military HIV Natural History Study were de-identified and anonymous. The Office of the Protection of Research Subjects at the Scripps Research Institute has reviewed and approved the research project described in this manuscript. A copy of the approval letter is provided in the supplementary material (S1 Text).

Plasma samples were obtained from 93 patients who had been treated with therapies that included protease inhibitors. For the samples sequenced in this study, all therapies were protease inhibitor (PI) based with a single PI (some with ritonavir (RTV) boosting), but included combinations of nucleoside and non-nucleoside reverse transcriptase inhibitors (NRTIs and NNRTIs). Therapies prior to sequencing were NRTI and NNRTI-based with no PIs. Although some patients were sequenced only once and others several times, there are only two relevant PI therapies for each patient: the first of which all patients failed prior to sequencing, and a second therapy on which patients were successfully treated or continued to fail treatment. Patients with multiple sequencing points after initial failure did not receive additional new therapies.

Samples were obtained when therapy failed to adequately suppress viral replication (>1,000 copies/mL), allowing multiple samples to be taken for some patients. Following extraction of viral RNA from these patient samples, 40 cycles of one-step RT-PCR was used to generate two 1 kilobase amplicons that spanned HIV Gag and protease. Primer design was based on conserved regions of the HIV-1 genome and follows the procedure in [22].

A liquid handling robot (Biomek NX, Beckman Coulter, Brea, CA, USA) was used to pool the two regions of amplified cDNA. Then, the pooled and amplified cDNA was prepared for sequencing using the NEBNext DNA Library Prep Master Mix Set (NEB, Ipswich, MA, USA). Specifically, the two amplicons were pooled in equimolar amounts, then 1ug of this mixture was fragmented to an average size of 275bp via mechanical shearing (S2 instrument, Covaris, Woburn, MA,USA). The samples were ligated to 1 of 48 sequencing adapters, each containing a unique 6bp index for downstream demultiplexing. (These adapters were custom made by ordering oligos from IDT. The sequences of the oligos are the same or similar to Illumina’s Trueseq indexes.) The fragments were size selected using Ampure beads (Beckman Coulter), and subsequently amplified using six cycles of PCR. After validating the libraries using the Bioanalyzer (Agilent, Santa Clara, CA, USA) and Qubit system (Life Technologies), the libraries were loaded onto the cBot (Illumina, San Diego, CA, USA), clonally amplified, and sequenced using 4 lanes on the Hiseq 2000 (Illumina) to yield an average of 4.3 million of 100bp paired end reads per sample.

After sequencing, reads were mapped to the Gag-Pol region of the HIV-1 HXB2 consensus sequence using Burrows-Wheeler Aligner (BWA, version 0.5.9-r16). The average mapping success rate to the HXB2 reference was 89.85%, with minimum, first quartile, and third quartile values of 54.72%, 86.99%, and 95.82%, respectively. Mapping results were corrected using indel recalibration and base quality score recalibration with Genome Analysis Toolkit (GATK, version 2.6-4-g3e5ff60). Reads with low recalibrated quality scores (MAPQ<30) were discarded. Single-site variants were called using VarScan (version 2.3). Samples with low coverage over either *gag* or protease were excluded from analysis. The average coverage over all positions in each sample is 202141 overlapping reads, with first and third quartile values of 152188 reads and 245360 reads, respectively, with the distribution of coverages skewed toward very deep coverage. The highest average coverage observed was 671075 reads, and the lowest average coverage was 26161 reads.

### Conventional sequence data

Variation in the deep sequencing data was compared to protease sequence variation in the Stanford HIV Database and Gag/Gag-Pol sequence variation in the Los Alamos National Laboratory HIV Sequence Database. For protease sequences, the 4/29/2013 downloadable protease dataset was downloaded from http://hivdb.stanford.edu/pages/geno-rx-datasets.html [24]. Non-ambiguous, complete subtype B sequences were separated into two sets of 4919 sequences exposed to treatment involving protease inhibitors and 12764 drug-naive sequences. Each entry in the downloadable dataset contains at least a nucleotide sequence or a list of amino acid substitutions. Entries with available nucleotide data were translated using IUPAC standard protein codes and, if any ambiguities existed in the translated sequence or nucleotide data was unavailable for that sequence, corresponding protein sequence data was used to fill in any ambiguities in the translated sequence.

Gag sequences were downloaded using the following settings through the standard search interface from http://www.hiv.lanl.gov/content/sequence/HIV/mainpage.html: *virus*: HIV-1; *subtype*: B; *culture method*: any; *Only drug naïve sequences*: checked; *genomic region*: Gag (searching with amino acid index 790–2292 results in identical results). The resulting 2378 Gag amino acid sequences were downloaded using squeeze gap handling and aligned to a HXB2 reference sequence. All but one sequence (which was discarded) had gaps and ambiguous amino acids at less than 10% of their length. Uncultured drug-naive gag sequences were also used; although the variation in the uncultured sequences is not identical to that in all naïve sequences (S4 Fig), due to the size of the uncultured set (<350 sequences), the full naïve set was used.

### Two-sample proportion test

To access the differences in variation between patients who failed therapy and patients who were successfully treated, we used the two sample proportion test. Patients were partitioned into two groups based on therapy: success and failure of sizes *N*_*s*_ and *N*_*f*_ respectively. In each patient, mutations present at or above 1% are considered detectable. For a given mutant at a single position, the proportion of patients with that mutation detectable is calculated in each group, *P*_*s*_ and *P*_*f*_. While the distribution of P_s_ and P_f_ are not necessarily normal, the distribution of (*P*_*f*_-*P*_*s*_) is normally distributed.

The pooled proportion *P*_*p*_

$$P_{p}=\frac{P_{s}\times N_{s}+P_{f}\times N_{f}}{N_{s}+N_{f}}$$

and the standard error

$$\text{SE}=\sqrt{P_{p}(1-P_{p})(\frac{1}{N_{s}}+\frac{1}{N_{f}})}$$

are computed. From these quantities, a z-score and p-value can be computed assuming a normal distribution using *Z* = (*P*_*f*_-*P*_*s*_)/SE. A p-value is computed for each mutation at all 599 positions for which the mutation is detectable in at least 5 patients who failed therapy. Statistically significant mutants were identified after correcting for multiple hypothesis testing using the Holm-Bonferroni method with family wise error rates of 0.1 and 0.01 (denoted with * and ** in Fig 3, respectively).

### Pairwise covariation

To assess the covariation amongst *gag* and protease mutations between a set of deep sequenced samples, we have constructed a protocol which estimates the joint probability of observing a double mutant and we use the mutual information (MI) to quantify how this probability deviates from a null model. For a particular pair of positions and given *N* samples, the probabilities of observing a mutation at position 1 and a mutation at position 2 individually in sample *i* are *p*_*X*_ and *p*_*Y*_, where 1≤*i*≤*N*. For each sample *i*, we then construct a 2×2 table of the joint probabilities of observing a double mutation (XY), only one mutation (X0, 0Y), and no mutations (00).

pXYpX0p0Yp00

These joint probabilities are constrained by the row marginal probabilities *p*_*X*_, 1-*p*_*X*_, and the column marginal probabilities *p*_*Y*_, 1-*p*_*Y*_ such that only one joint probability is free. Take this to be the double mutation probability *p*_*XY*_, which is bounded such that:

$$\text{max}(0,p_{X}+p_{Y}-1)\leq p_{XY}\leq \text{min}(p_{X},p_{Y})$$

Moreover, the bounds are exact given that for any number *q* between the upper and lower bounds, there exists a valid 2×2 table of probabilities with *p*_*XY*_ equal to *q*. Note that if *p*_*X*_ and *p*_*Y*_ are close to either 0 or 1, then the bounds become very tight. This property is particularly useful in our analysis of the *gag* and protease deep sequencing data.

The bounds on *p*_*XY*_ are computed for each sample i, and are then averaged yielding a single upper and lower bound for the average double mutation probability. Using the average single site probabilities *p*_*X*_ and *p*_*Y*_, we construct a full 2×2 probability table for each of the averaged bounds:

pXYlowerpX0lowerp0Ylowerp00lowerpXYupperpX0upperp0Yupperp00upper

We also construct an estimate of the joint probabilities assuming X and Y mutate independently, such that

pXY0pX00p0Y0p000=pXpYpX(1-pY)(1-pX)pY(1-pX)(1-pY)

The deviation of the average 2×2 probability tables from this independent table represents the covariation of X and Y. For assessing positive covariation, the most conservative estimate of *p*_*XY*_ is given by $\text{max}(0,p_{XY}^{lower})$, whereas the least conservative estimate of *p*_*XY*_ is given by $p_{XY}^{upper}$. For assessing negative covariation the most conservative estimate of *p*_*XY*_ is given by $\text{min}(0,p_{XY}^{upper})$, whereas the least conservative estimate of *p*_*XY*_ is given by $p_{XY}^{lower}$. The mutual information is defined as

$$\text{MI}=\sum_{a\in \{X,0\},b\in \{Y,0\}}p_{ab}\text{log}(p_{ab}/p_{ab}^{0})$$

It is easily shown that MI = 0 when $p_{XY}=p_{XY}^{0}$, and increases as *p*_*XY*_ moves away from $p_{XY}^{0}$ in either direction. Given our bounding procedure, the most positively correlated pairs of mutations are those with the largest MI. Using the deep sequencing data, this procedure is conducted for all pairs of mutations for which the frequencies of the mutations are above 1% in 5 or more samples.

It is important to understand the relationship between the proposed procedure for deep sequenced data and those for MSA data consisting of binary counts from single sequences. Each sequence in a MSA provides a single count for a particular single mutant for each position (double mutant for each pair of positions). The counts from all sequences are averaged to get one- and two-site frequency counts, which approximate the univariate and bivariate marginal probabilities when the number of sequences is very large. In contrast, each patient sample in the deep sequenced data essentially provides a MSA of several million sequences. When aggregated, we calculate the mean univariate and bivariate probabilities from each sample, not counts. Therefore, although our dataset contains many fewer samples than sequences in a MSA, a sample in the deep sequenced data provides considerably more information than a single sequence in a MSA.

The permutation procedure used to generate p-values for the Jaccard similarity coefficient for MSA data in [3], if the number of permutations tends to infinity, is equivalent to computing Fisher's exact test (See S8 Table). This procedure determines how likely the observed data could arise from chance and is not a direct measure of the correlation between two mutations. For instance, with this procedure it is possible to know a pair of mutations is weakly correlated with high confidence.

Moreover, for MSA data, ranking the strength of correlation by mutual information for a 2×2 table of probabilities is equivalent to ranking by the log likelihood ratio statistic (LR) for testing independence in a 2×2 table of counts, because LR = -*N*×MI. But, as the total sample size tends to infinity, ranking based on LR is asymptotically equivalent to ranking based on Fisher’s exact test of independence [64]. Therefore, the proposed procedure for deep sequencing data differs from previous analyses of MSA data [3] mainly in the necessary step of constructing lower and upper probability tables and, to a lesser extent, in the use of mutual information for ranking correlations in probability tables without depending on the total sample size.

Alternative analyses to determine correlations were attempted without estimating joint probabilities, such as using Kendall's tau. In this analysis method, for each mutant in a pair of residues, a vector of single-site frequencies is constructed from the frequencies in each sample. Kendall's tau-b for each such pair is calculated with an accompanying z-score from which a p-value can be calculated. However, Kendall's tau is not appropriate for this type of data because Kendall's tau typically requires the underlying population to be bivariate normal; the frequencies we observe are not normally distributed. Furthermore, Kendall's tau is extremely sensitive to data located at the maximum or minimum of the possible spectrum of values, and the fact that many mutations are either mostly absent or dominant in a single sample produce unreliable results with Kendall's tau.

### Phylogenetic correction to MI

We recognize that the Mutual Information (MI) does not account for correlations which arise from phylogenetic relationships among the population of interest. In this specific study, where there is the population within each patient and the combined population of all patients, any phylogenetic correction to MI will only reduce phylogenetic influence in the combined population. Alongside of the uncorrected MI, we have computed MIp [65]. Prior analysis of many methods developed to account for phylogeny, not limited to MI based statistics, has found MIp to be the leading choice for large datasets [66]. We find that the two statistics, MI and MIp, give similar rankings of the most correlated pairs of PR-PR residues as shown in S6 Fig. Shown in S7 Fig is the recovery of positively correlated PR-PR pairs identified in [3] using MIp and the recovery is similar to that using MI shown in Fig 5. Although we lack the sequence linkage to apply other simple corrections for phylogenetic effects, such as sequence reweighting [55,57,59], MI of weighted and unweighted HIV sequences has been shown to be similar [55]. Because results between MI and MIp were found to be similar, we used the uncorrected MI throughout the study.

### All-atom distance calculation

For pairs of residues in MA, CA, NC, and PR, the smallest all-atom distances were calculated by scanning through PDB files for all possible multimerizations of each protein: MA (monomer PDB 2H3F*, trimer PDB 1HIW), CA (monomer PDB 3MGE, dimer PDB 2M8L*, pentamer PDB 3P05, hexamer PDB 3MGE), NC (monomer PDB 2EXF*), PR (dimer PDB 1ODW). For a single pair, the distance between all combinations of heavy atoms (backbone and sidechain) was computed for each combination of chains (for multimers) for each conformation. The minimum distance (*R*_*ij*_) is listed in Table 5, S6 Table, as well as the pair of chains, atoms, and the PDB structure from which the distance is derived. Pairs with smallest *R*_*ij*_ when on different chains are denoted with the chain combination; otherwise, the chain combination is listed as '-'. For structures derived from NMR data (denoted with * above), the smallest atom-atom distance was calculated for each model, and the smallest distance from all models is reported in Table 5. The distribution of model distances for each PDB derived from NMR is listed in S7 Table.

## Supporting Information

S1 Fig Protease mutation patterns in PI-experienced samples. Shown is the distribution of mutation counts in drug naive sequences (blue), sequences from patients treated with 1 protease inhibitor (red), and sequences from patients treated with 2 or more protease inhibitors (green) [27]. Shown in black is the distribution of fixed mutations (mutations with frequencies greater than 98% in a single sample) found in our deep sequenced samples.
(TIFF)

S2 Fig Samples with variants vs sequence position over PR. Bar charts representing the number of samples in which amino acid variants are observed at each position in protease derived from deep sequencing (gray), 12,759 PI-naive subtype B protease sequences from Stanford HIVDB (blue), and 4,919 PI-experienced subtype B protease sequences from Stanford HIVDB (red); for sequence details, see http://hivdb.stanford.edu/modules/lookUpFiles/geno-rx-datasets/PR.txt. Variants shown from deep sequencing occur at frequencies above 1% in 5 or more patients and variants shown from HIVDB are present in at least 1% of sequences. Positions at which the variation between the two datasets is small (|*f*_DS_-*f*_HIVDB_|<10%) are faded.
(TIFF)

S3 Fig Recovery of correlated protease-protease pairs. Shown is a plot of the precision for the top 5% of correlated PR-PR pairs ranked by mutual information using both the lower (left) and upper (right) bound on the double mutant probability. As in Fig 5, shown are the top 5% of 1275 pairs with 127 putative true positives from Stanford HIVDB.
(TIFF)

S4 Fig Conservation of LANL drug-naive sequences. Shown are several plots of conservation index (CI) [28] versus Gag sequence position for two different sets of drug-naive sequences from the Los Alamos HIV sequence database: a set of 342 uncultured, drug-naive sequences, and a set of 2384 drug-naive sequences. (Top) The two datasets have similar CI over nucleocapsid. (Middle, Bottom) However, at many Gag positions, the conservation index varies greatly (up to 140%) between the two datasets.
(TIFF)

S5 Fig Estimated bounds on double mutant probability per sample. Shown in each panel are the known double mutant bivariate marginal probability (red square) and the estimated lower and upper bounds on the probability shown as error bars in all samples for a given pair. Samples on the x-axis are sorted by the magnitude of the known double mutant probability in that sample, and the order of the samples is not necessarily the same for each panel.
(TIFF)

S6 Fig APC correction to MI versus MI. Shown is the ranking of 25 PR-PR pairs ranked by MIp [64] vs the uncorrected MI.
(TIFF)

S7 Fig Recovery of correlated protease-protease pairs using MIp. Shown is a plot of the precision for the top 5% of correlated PR-PR pairs ranked by MIp [64]. As in Fig 5 and S3 Fig, shown are the top 5% of 1275 pairs with 127 putative true positives from Stanford HIVDB.
(TIFF)

S1 Text Letter of approval for proper handling of patient information and serum. This letter from The Scripps Research Institute Office for the Protection of Research Subjects shows approval of the research project described in this article.
(PDF)

S1 Table Observed Gag cleavage site mutations. ^a^Blank entries indicate mutant was not present with 98% frequency or greater in any sample. ^b^Blank entries indicate mutant was has not been associated with PI-exposure or-resistance (reported in [6]).
(DOC)

S2 Table Observed Gag cleavage site mutants which are variants of PI-associated mutations. Observed cleavage site mutants which occur at PI-associated residues are indicated in bold. aBlank entries indicate mutant was not present with 98% frequency or greater in any sample bBlank entries indicate mutant was has not been associated with PI-exposure or-resistance (reported in [6]).
(DOC)

S3 Table Top 5% of most strongly correlated pairs of PR-PR positions. (DOC)

S4 Table Top 1% of most strongly correlated pairs of Gag-PR positions. †Due to Gag-Pol frameshifting, Gag codons 499–500 code protease residue 12. Simultaneous synonymous nucleotide substitutions at Gag 499 and nonsynonymous nucleotide substitutions at Gag 500 result in mutation in protease residue 12. We therefore observe a strong correlation between Gag 500 and PR 12, though this correlation holds little co-evolution information because both amino acid mutations are the manifestation of one set nucleotide substitutions. We do not observe strong Gag-PR correlations in the frameshift region (Gag 488–500 and PR 1–12), likely due to the observed conservation of PR residues 1–9 and 11.
(DOC)

S5 Table Top 1% of most strongly correlated pairs of Gag-Gag positions. (DOC)

S6 Table Smallest all-atom distances between strongly correlated pairs of residues in Gag and protease as identified by mutual information. Listed are the smallest atom-atom distances (only heavy atoms, excluding side chains) for the most strongly correlated pairs of residues from the three regions PR-PR, Gag-PR, Gag-Gag ranked by MI in representative structures. For structures with multiple chains, inter-chain distances were computed and the chain and atom combinations of the smallest calculated distance for each pair are listed. Pairs with atom-atom distances above 8Å are listed in gray. ^a^MA: matrix monomer, PDB 2H3F; MA3: matrix trimer, PDB 1HIW; CA: capsid monomer, PDB 3MGE; CA2: capsid dimer, PDB 2M8L; CA5: capsid pentamer, PDB 3P05; CA6: capsid hexamer, PDB 3MGE; NC: nucleocapsid monomer, PDB 2EXF; PR2: protease dimer, PDB 1ODW. ^b^For PDB files with multiple structural models, the model number with the smallest atom-atom distance is listed.
(DOC)

S7 Table Distribution of atom-atom distances among models in PDBs derived from NMR. Some of the distance calculations used in Tables 5 and S6 are derived from ensembles of structures. Shown here are the minimum, mean, and maximum atom-atom distances for all affected position pairs calculated from each PDB with an ensemble of structural models. For distances <8Å, we find there to be little variability across structural models.
(DOC)

S8 Table PR-PR pairs ranked by Fisher exact test p-value calculated from 2013 HIVDB sequences. We show a comparison between PR-PR pair rankings calculated using Fisher’s exact test on a MSA provided by the Stanford HIVDB [24] dated 4/29/2013 and the permutation test presented in [3].
(DOC)
