# Supplementary figures and images for: Deep Sequencing of Protease Inhibitor Resistant HIV Patient Isolates Reveals Patterns of Correlated Mutations in Gag and Protease
Source: PLoS Comput Biol. 2015 Apr 20;11(4):e1004249. doi: 10.1371/journal.pcbi.1004249 (PMC4404092; doi:10.1371/journal.pcbi.1004249)

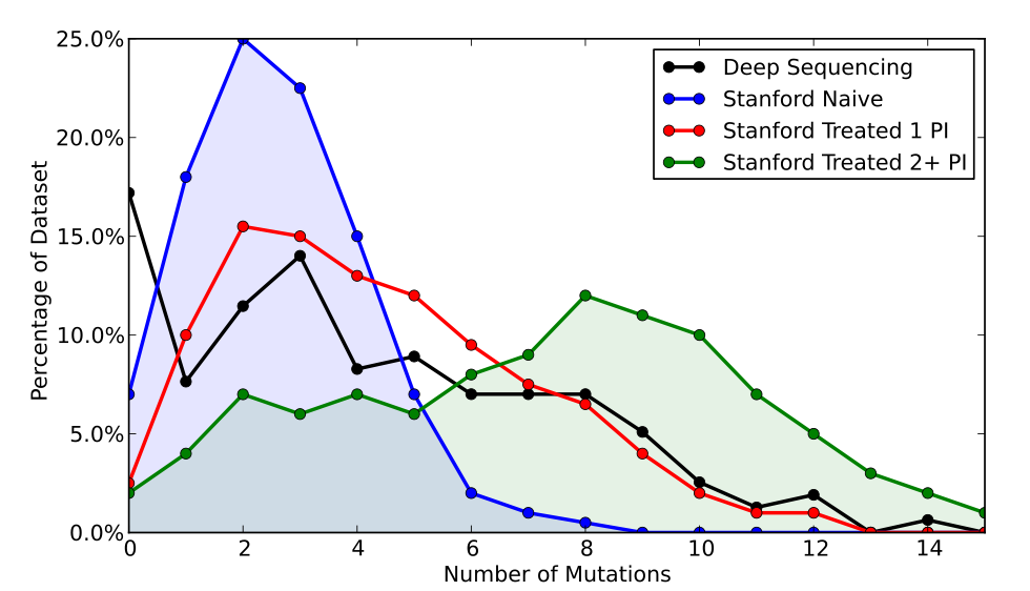

Supplement: S1 Fig — Shown is the distribution of mutation counts in drug naive sequences (blue), sequences from patients treated with 1 protease inhibitor (red), and sequences from patients treated with 2 or more protease inhibitors (green) [27]. Shown in black is the distribution of fixed mutations (mutations with frequencies greater than 98% in a single sample) found in our deep sequenced samples. (TIFF) [file pcbi.1004249.s001.tiff]

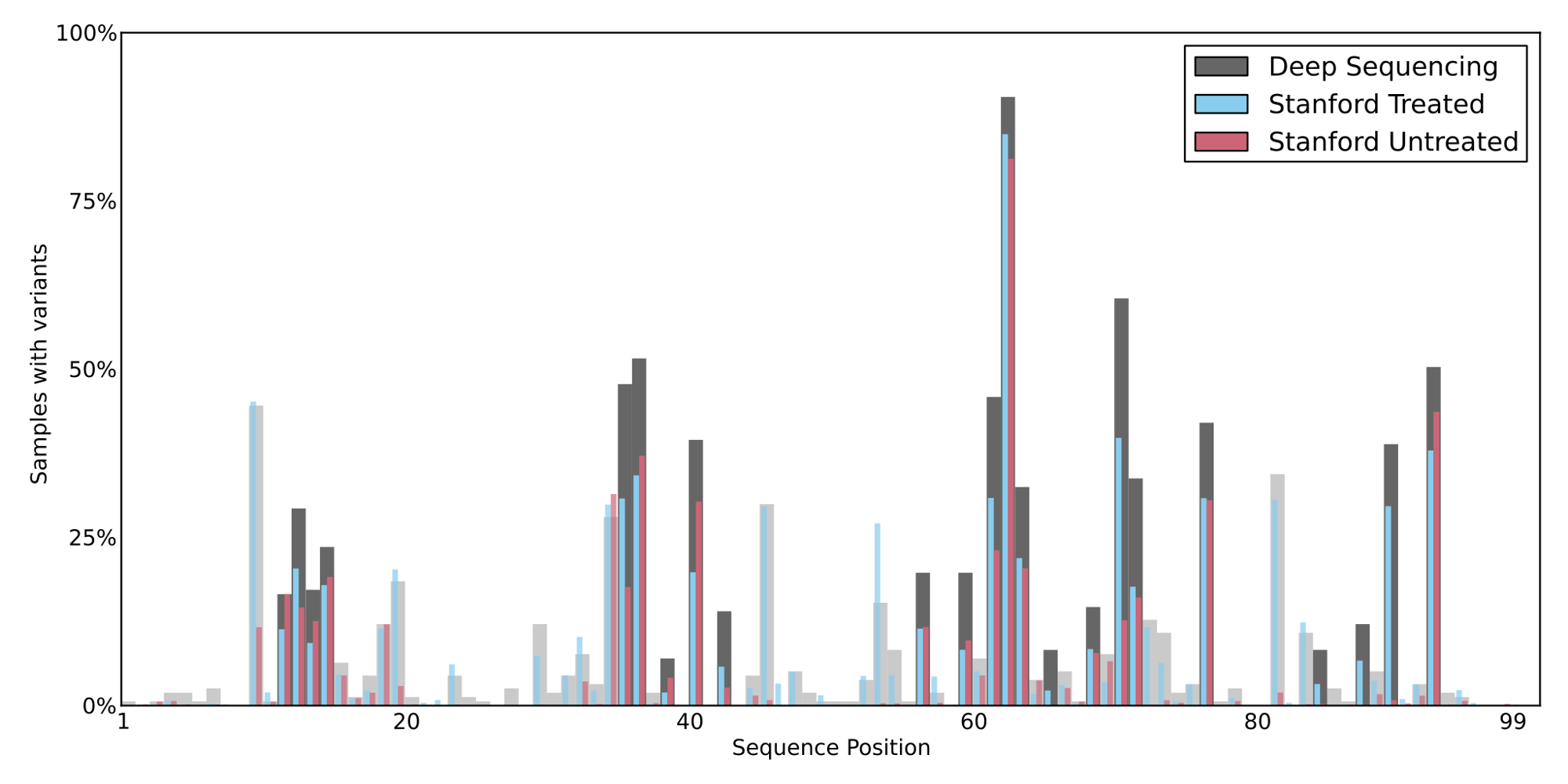

Supplement: S2 Fig — Bar charts representing the number of samples in which amino acid variants are observed at each position in protease derived from deep sequencing (gray), 12,759 PI-naive subtype B protease sequences from Stanford HIVDB (blue), and 4,919 PI-experienced subtype B protease sequences from Stanford HIVDB (red); for sequence details, see http://hivdb.stanford.edu/modules/lookUpFiles/geno-rx-datasets/PR.txt. Variants shown from deep sequencing occur at frequencies above 1% in 5 or more patients and variants shown from HIVDB are present in at least 1% of sequences. Positions at which the variation between the two datasets is small (|f DS-f HIVDB|<10%) are faded. (TIFF) [file pcbi.1004249.s002.tiff]

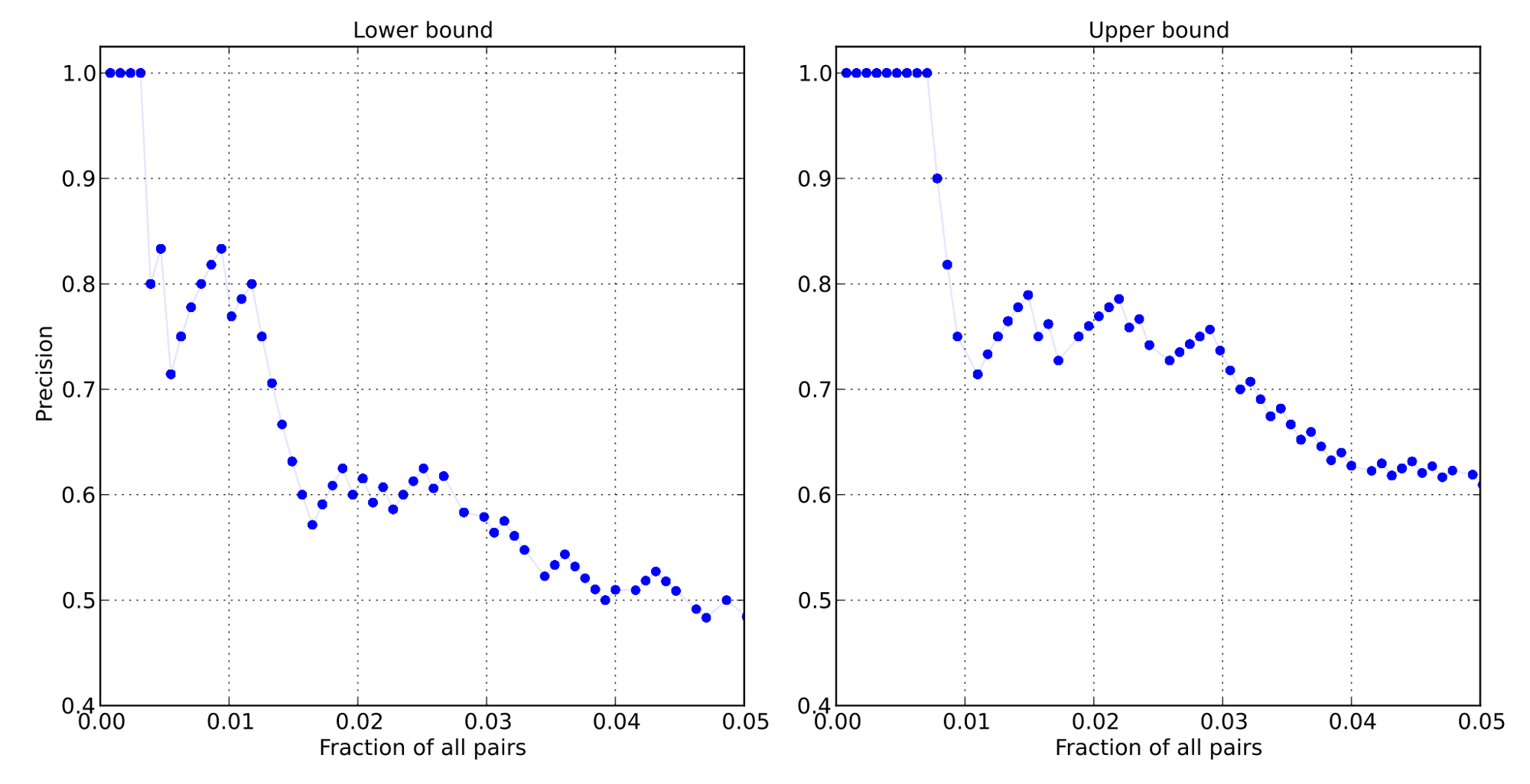

Supplement: S3 Fig — Shown is a plot of the precision for the top 5% of correlated PR-PR pairs ranked by mutual information using both the lower (left) and upper (right) bound on the double mutant probability. As in Fig 5, shown are the top 5% of 1275 pairs with 127 putative true positives from Stanford HIVDB. (TIFF) [file pcbi.1004249.s003.tiff]

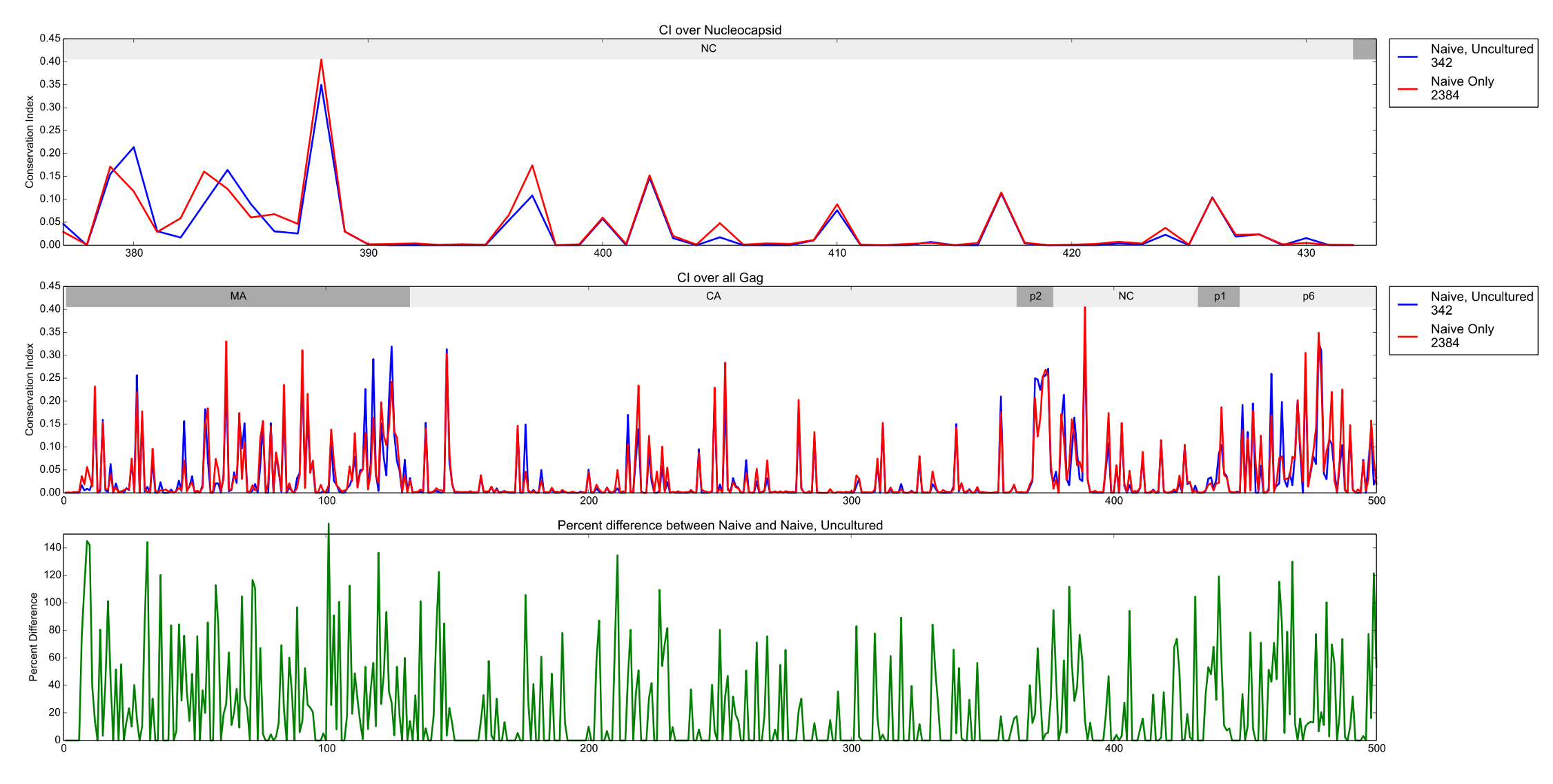

Supplement: S4 Fig — Shown are several plots of conservation index (CI) [28] versus Gag sequence position for two different sets of drug-naive sequences from the Los Alamos HIV sequence database: a set of 342 uncultured, drug-naive sequences, and a set of 2384 drug-naive sequences. (Top) The two datasets have similar CI over nucleocapsid. (Middle, Bottom) However, at many Gag positions, the conservation index varies greatly (up to 140%) between the two datasets. (TIFF) [file pcbi.1004249.s004.tiff]

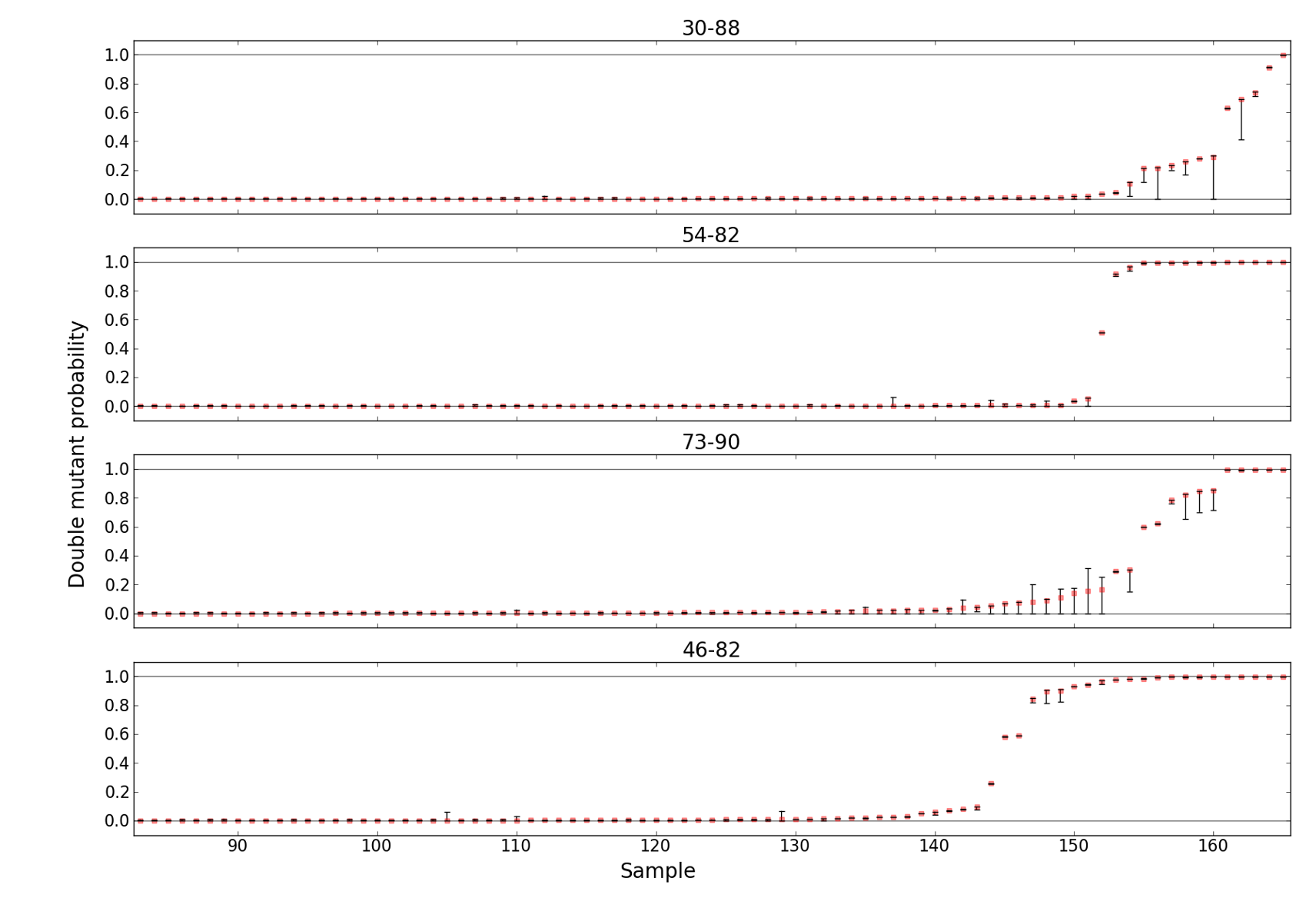

Supplement: S5 Fig — Shown in each panel are the known double mutant bivariate marginal probability (red square) and the estimated lower and upper bounds on the probability shown as error bars in all samples for a given pair. Samples on the x-axis are sorted by the magnitude of the known double mutant probability in that sample, and the order of the samples is not necessarily the same for each panel. (TIFF) [file pcbi.1004249.s005.tiff]

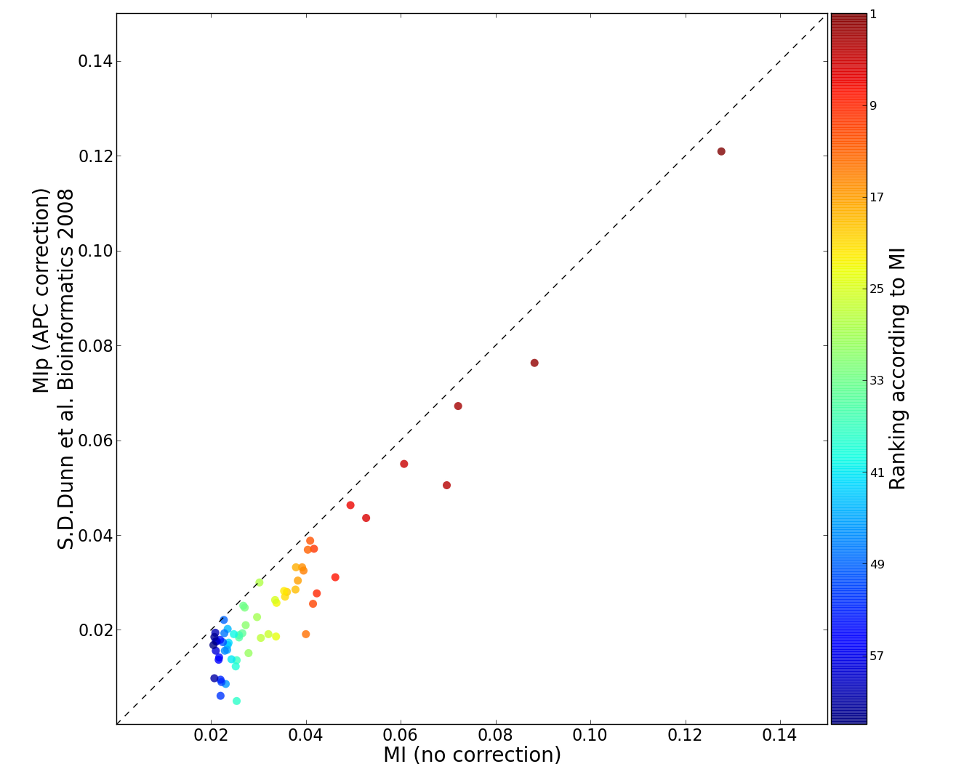

Supplement: S6 Fig — Shown is the ranking of 25 PR-PR pairs ranked by MIp [64] vs the uncorrected MI. (TIFF) [file pcbi.1004249.s006.tiff]

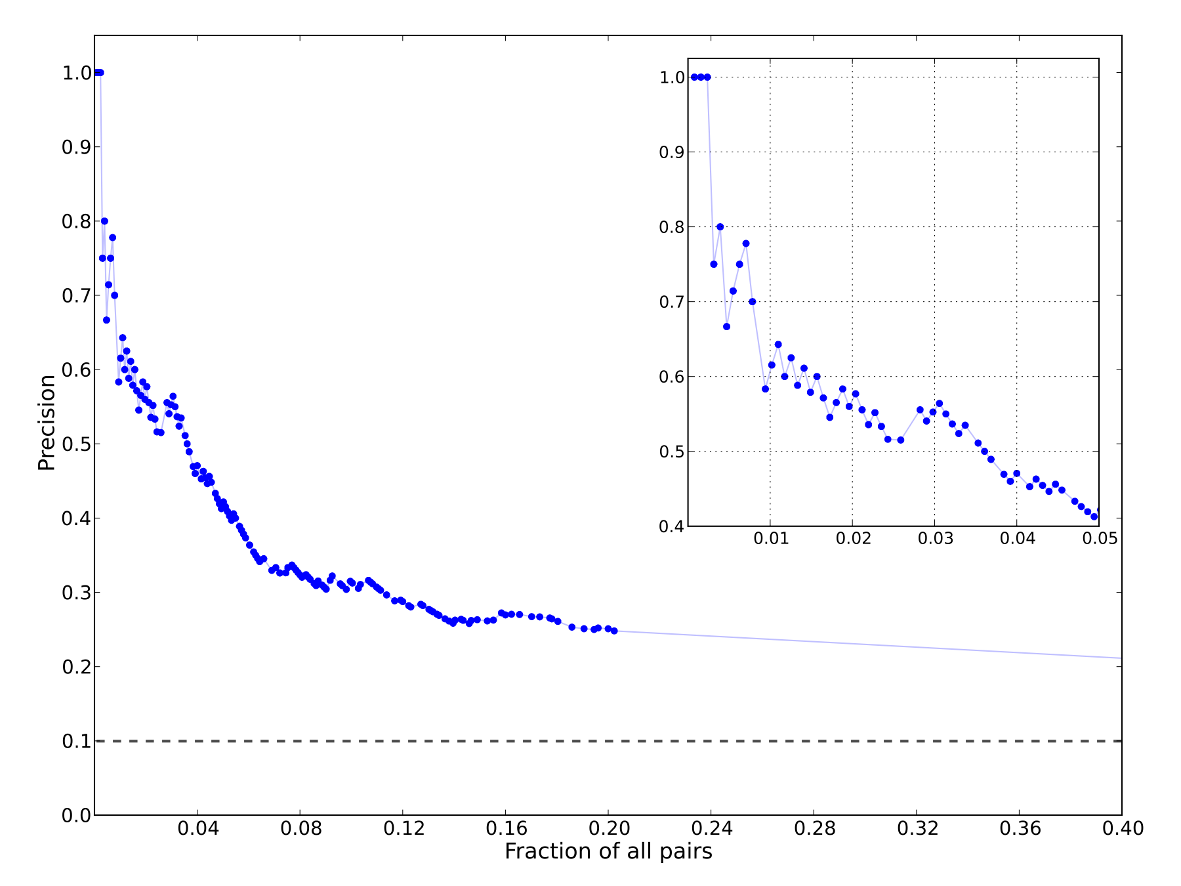

Supplement: S7 Fig — Shown is a plot of the precision for the top 5% of correlated PR-PR pairs ranked by MIp [64]. As in Fig 5 and S3 Fig, shown are the top 5% of 1275 pairs with 127 putative true positives from Stanford HIVDB. (TIFF) [file pcbi.1004249.s007.tiff]
