# Supplementary material for: Deep Sequencing of Protease Inhibitor Resistant HIV Patient Isolates Reveals Patterns of Correlated Mutations in Gag and Protease
Source: PLoS Comput Biol. 2015 Apr 20;11(4):e1004249. doi: 10.1371/journal.pcbi.1004249 (PMC4404092; doi:10.1371/journal.pcbi.1004249)
Supplement: S1 Text — This letter from The Scripps Research Institute Office for the Protection of Research Subjects shows approval of the research project described in this article. (PDF) [file pcbi.1004249.s008.pdf]

Scripps
Office for the Protection of Research Subjects

Scripps IRB

11025 North Torrey Pines Road
Suite 200
La Jolla, CA 92037

Approval Notice

Investigator: Bruce Torbett, PhD, MSPH

Department: Molecular & Experimental Medicine

Approved Research Sites: The Scripps Research Institute and Uniformed Services University of the Health Sciences

Project Title: HIV Macromolecular Interactions and Impact on Viral Evolution of Drug Resistance

Protocol No: IRB-12-5929

Risk Category: Minimal

Type of Review: Expedited-Continuing

Your research project indicated above was reviewed and approved by an IRB officer on the review date stamped below. Approval expires ***12 months*** from this date.

Approval carries with it the understanding that you will inform the Committee promptly should a serious adverse reaction occur, and that you will make no modification to the protocol or consent form (if applicable) without prior IRB approval.

The IRB may suspend or terminate the approval of research that is not conducted in accordance with the requirements set forth by the committee or that has been associated with unexpected serious harm to subjects.

Thank you for your cooperation.

(Normal Blood Donors. Use of stored serum from HIV-1 positive samples from IDCRP of the Uniformed Services University of the Health Sciences)

Signature applied by Ronald A. Simon on 04/28/2014 01:37:07 PM PDT

IRB Officer
