# Supplementary material for: Deep Sequencing of Protease Inhibitor Resistant HIV Patient Isolates Reveals Patterns of Correlated Mutations in Gag and Protease
Source: PLoS Comput Biol. 2015 Apr 20;11(4):e1004249. doi: 10.1371/journal.pcbi.1004249 (PMC4404092; doi:10.1371/journal.pcbi.1004249)
Supplement: S1 Table — aBlank entries indicate mutant was not present with 98% frequency or greater in any sample. bBlank entries indicate mutant was has not been associated with PI-exposure or-resistance (reported in [6]). (DOC) [file pcbi.1004249.s009.doc]

**Table S1:** Observed Gag cleavage site mutations

| **Cleavage Site** | **Mutation** | **Samples Observed** | **Samples Fixed^a^** | **Associated with PI Exposure/ Resistance^b^** |
| --- | --- | --- | --- | --- |
| MA/CA | V128I | 14 |  | Yes/Yes |
|  | V128A | 9 |  | Yes/Yes |
|  | Q130H | 6 |  |  |
|  | N131S | 23 |  |  |
|  | Y132F | 18 | 5 | Yes/No |
| CA/p2 | R361K | 6 |  |  |
|  | V362I | 19 | 7 | Yes/No |
|  | S368C | 5 |  | Yes/No |
| p2/NC | S373A | 10 |  |  |
|  | S373P | 79 | 48 | Yes/No |
|  | A374N | 26 | 20 |  |
|  | A374P | 15 | 10 | Yes/No |
|  | A374S | 5 |  | Yes/No |
|  | A374T | 41 | 7 |  |
|  | T375A | 53 | 27 |  |
|  | T375N | 25 | 12 | Yes/No |
|  | T375S | 7 |  | Yes/No |
|  | I376M | 6 |  |  |
|  | I376V | 34 | 10 | Yes/No |
|  | M378I | 38 | 10 |  |
|  | M378V | 13 |  |  |
|  | R380G | 10 |  |  |
|  | R380K | 93 | 46 |  |
|  | G381S | 7 |  | Yes/No |
|  | N382G | 5 |  |  |
|  | N382K | 6 |  |  |
| NC/p1 | E428G | 9 |  | Yes/No |
|  | A431V | 41 | 15 | Yes/Yes |
|  | K436R | 22 |  | Yes/Yes |
|  | I437V | 19 |  | Yes/Yes |
| p1/p6 | R444G | 6 |  |  |
|  | L449F | 10 |  | Yes/Yes |
|  | L449P | 11 | 6 | Yes/Yes |
|  | S451N | 57 | 10 |  |
|  | R452K | 7 |  | Yes/Yes |
|  | P453L | 36 | 9 | Yes/Yes |
|  | P453S | 8 |  |  |
|  | P453T | 13 |  | Yes/Yes |
