# Supplementary material for: Deep Sequencing of Protease Inhibitor Resistant HIV Patient Isolates Reveals Patterns of Correlated Mutations in Gag and Protease
Source: PLoS Comput Biol. 2015 Apr 20;11(4):e1004249. doi: 10.1371/journal.pcbi.1004249 (PMC4404092; doi:10.1371/journal.pcbi.1004249)
Supplement: S2 Table — Observed cleavage site mutants which occur at PI-associated residues are indicated in bold. aBlank entries indicate mutant was not present with 98% frequency or greater in any sample bBlank entries indicate mutant was has not been associated with PI-exposure or-resistance (reported in [6]). (DOC) [file pcbi.1004249.s010.doc]

**Table S2:** Observed Gag cleavage site mutants which are variants of PI-associated mutations

| **Cleavage Site** | **Mutation** | **Samples Observed** | **Samples Fixed^a^** | **Associated with PI Exposure/ Resistance^b^** |
| --- | --- | --- | --- | --- |
| p2/NC | **S373A** | **10** |  |  |
|  | S373P | 79 | 48 | Yes/No |
|  | **A374N** | **26** | **20** |  |
|  | A374P | 15 | 10 | Yes/No |
|  | A374S | 5 |  | Yes/No |
|  | **A374T** | **41** | **7** |  |
|  | **T375A** | **53** | **27** |  |
|  | T375N | 25 | 12 | Yes/No |
|  | T375S | 7 |  | Yes/No |
|  | **I376M** | **6** |  |  |
|  | I376V | 34 | 10 | Yes/No |
| p1/p6 | P453L | 36 | 9 | Yes/Yes |
|  | **P453S** | **8** |  |  |
|  | P453T | 13 |  | Yes/Yes |
