# Supplementary material for: Deep Sequencing of Protease Inhibitor Resistant HIV Patient Isolates Reveals Patterns of Correlated Mutations in Gag and Protease
Source: PLoS Comput Biol. 2015 Apr 20;11(4):e1004249. doi: 10.1371/journal.pcbi.1004249 (PMC4404092; doi:10.1371/journal.pcbi.1004249)
Supplement: S3 Table — (DOC) [file pcbi.1004249.s011.doc]

**Table S3:** Top 5% of most strongly correlated pairs of PR-PR positions

| **Protease Position 1** | **Protease Position 2** | **Observed in HIVDB** | **Position 1**  **PI-association** | **Position 2**  **PI-association** | **MI** | **P_xy_** | **P_xy_^0^** | **P_x_** | **P_y_** |
| --- | --- | --- | --- | --- | --- | --- | --- | --- | --- |
| 30 | 88 | Yes | Yes | Yes | 0.128 | 0.056 | 0.006 | 0.072 | 0.082 |
| 54 | 82 | Yes | Yes | Yes | 0.088 | 0.072 | 0.018 | 0.088 | 0.200 |
| 73 | 90 | Yes | Yes | Yes | 0.072 | 0.055 | 0.013 | 0.064 | 0.201 |
| 46 | 82 | Yes | Yes | Yes | 0.070 | 0.096 | 0.034 | 0.170 | 0.200 |
| 24 | 74 | No | Yes | Yes | 0.061 | 0.022 | 0.001 | 0.027 | 0.045 |
| 35 | 36 | Yes | Yes | Yes | 0.053 | 0.096 | 0.040 | 0.204 | 0.195 |
| 69 | 84 | No | No | Yes | 0.049 | 0.023 | 0.002 | 0.040 | 0.050 |
| 24 | 46 | Yes | Yes | Yes | 0.046 | 0.026 | 0.005 | 0.027 | 0.170 |
| 24 | 82 | Yes | Yes | Yes | 0.042 | 0.027 | 0.005 | 0.027 | 0.200 |
| 13 | 33 | Yes | Yes | Yes | 0.042 | 0.034 | 0.008 | 0.174 | 0.043 |
| 10 | 93 | Yes | Yes | Yes | 0.042 | 0.156 | 0.094 | 0.282 | 0.334 |
| 12 | 19 | Yes | No | No | 0.041 | 0.031 | 0.005 | 0.088 | 0.061 |
| 33 | 66 | No | Yes | Yes | 0.040 | 0.017 | 0.001 | 0.043 | 0.026 |
| 10 | 46 | Yes | Yes | Yes | 0.040 | 0.098 | 0.048 | 0.282 | 0.170 |
| 32 | 82 | Yes | Yes | Yes | 0.040 | 0.027 | 0.006 | 0.028 | 0.200 |
| 24 | 64 | No | Yes | No | 0.039 | 0.024 | 0.004 | 0.027 | 0.140 |
| 37 | 63 | No | No | Yes | 0.038 | 0.284 | 0.233 | 0.309 | 0.752 |
| 33 | 60 | No | Yes | Yes | 0.038 | 0.027 | 0.004 | 0.043 | 0.101 |
| 41 | 93 | No | No | Yes | 0.038 | 0.143 | 0.085 | 0.256 | 0.334 |
| 30 | 35 | No | Yes | Yes | 0.036 | 0.046 | 0.015 | 0.072 | 0.204 |
| 35 | 88 | No | Yes | Yes | 0.036 | 0.050 | 0.017 | 0.204 | 0.082 |
| 32 | 46 | Yes | Yes | Yes | 0.035 | 0.025 | 0.005 | 0.028 | 0.170 |
| 20 | 62 | Yes | Yes | Yes | 0.034 | 0.057 | 0.022 | 0.087 | 0.253 |
| 63 | 93 | Yes | Yes | Yes | 0.034 | 0.301 | 0.251 | 0.752 | 0.334 |
| 41 | 72 | No | No | Yes | 0.034 | 0.084 | 0.040 | 0.256 | 0.157 |
| 10 | 54 | Yes | Yes | Yes | 0.032 | 0.059 | 0.025 | 0.282 | 0.088 |
| 41 | 63 | No | No | Yes | 0.031 | 0.235 | 0.192 | 0.256 | 0.752 |
| 16 | 39 | Yes | No | No | 0.030 | 0.012 | 0.001 | 0.023 | 0.027 |
| 35 | 84 | No | Yes | Yes | 0.030 | 0.035 | 0.010 | 0.204 | 0.050 |
| 63 | 90 | Yes | Yes | Yes | 0.028 | 0.188 | 0.151 | 0.752 | 0.201 |
| 77 | 93 | Yes | Yes | Yes | 0.027 | 0.136 | 0.087 | 0.261 | 0.334 |
| 84 | 85 | Yes | Yes | Yes | 0.027 | 0.013 | 0.001 | 0.050 | 0.023 |
| 61 | 67 | No | No | No | 0.027 | 0.012 | 0.001 | 0.033 | 0.023 |
| 20 | 36 | Yes | Yes | Yes | 0.027 | 0.046 | 0.017 | 0.087 | 0.195 |
| 36 | 88 | No | Yes | Yes | 0.026 | 0.044 | 0.016 | 0.195 | 0.082 |
| 30 | 36 | No | Yes | Yes | 0.026 | 0.040 | 0.014 | 0.072 | 0.195 |
| 63 | 72 | No | Yes | Yes | 0.025 | 0.150 | 0.118 | 0.752 | 0.157 |
| 10 | 82 | Yes | Yes | Yes | 0.025 | 0.098 | 0.056 | 0.282 | 0.200 |
| 10 | 41 | No | Yes | No | 0.025 | 0.117 | 0.072 | 0.282 | 0.256 |
| 35 | 37 | Yes | Yes | No | 0.025 | 0.106 | 0.063 | 0.204 | 0.309 |
| 24 | 62 | No | Yes | Yes | 0.024 | 0.024 | 0.007 | 0.027 | 0.253 |
| 24 | 77 | No | Yes | Yes | 0.024 | 0.024 | 0.007 | 0.027 | 0.261 |
| 62 | 84 | No | Yes | Yes | 0.024 | 0.035 | 0.013 | 0.253 | 0.050 |
| 13 | 67 | No | Yes | No | 0.024 | 0.019 | 0.004 | 0.174 | 0.023 |
| 62 | 71 | Yes | Yes | Yes | 0.023 | 0.123 | 0.078 | 0.253 | 0.310 |
| 10 | 62 | Yes | Yes | Yes | 0.023 | 0.115 | 0.071 | 0.282 | 0.253 |
| 54 | 72 | No | Yes | Yes | 0.023 | 0.039 | 0.014 | 0.088 | 0.157 |
| 20 | 76 | No | Yes | Yes | 0.023 | 0.011 | 0.001 | 0.087 | 0.014 |
| 14 | 70 | No | No | No | 0.023 | 0.017 | 0.003 | 0.053 | 0.046 |
| 12 | 90 | No | No | Yes | 0.023 | 0.044 | 0.018 | 0.088 | 0.201 |
| 10 | 36 | Yes | Yes | Yes | 0.022 | 0.094 | 0.055 | 0.282 | 0.195 |
| 10 | 24 | Yes | Yes | Yes | 0.022 | 0.024 | 0.008 | 0.282 | 0.027 |
| 36 | 63 | No | Yes | Yes | 0.022 | 0.179 | 0.147 | 0.195 | 0.752 |
| 32 | 71 | Yes | Yes | Yes | 0.022 | 0.025 | 0.009 | 0.028 | 0.310 |
| 20 | 90 | Yes | Yes | Yes | 0.022 | 0.044 | 0.018 | 0.087 | 0.201 |
| 35 | 71 | No | Yes | Yes | 0.022 | 0.103 | 0.063 | 0.204 | 0.310 |
| 35 | 85 | No | Yes | Yes | 0.021 | 0.019 | 0.005 | 0.204 | 0.023 |
| 13 | 88 | No | Yes | Yes | 0.021 | 0.038 | 0.014 | 0.174 | 0.082 |
| 37 | 77 | No | No | Yes | 0.021 | 0.123 | 0.081 | 0.309 | 0.261 |
| 69 | 85 | No | No | Yes | 0.021 | 0.011 | 0.001 | 0.040 | 0.023 |
| 63 | 71 | Yes | Yes | Yes | 0.021 | 0.272 | 0.233 | 0.752 | 0.310 |
| 60 | 61 | Yes | Yes | No | 0.021 | 0.018 | 0.003 | 0.101 | 0.033 |
| 66 | 71 | No | Yes | Yes | 0.021 | 0.023 | 0.008 | 0.026 | 0.310 |
