# Supplementary material for: Deep Sequencing of Protease Inhibitor Resistant HIV Patient Isolates Reveals Patterns of Correlated Mutations in Gag and Protease
Source: PLoS Comput Biol. 2015 Apr 20;11(4):e1004249. doi: 10.1371/journal.pcbi.1004249 (PMC4404092; doi:10.1371/journal.pcbi.1004249)
Supplement: S4 Table — †Due to Gag-Pol frameshifting, Gag codons 499–500 code protease residue 12. Simultaneous synonymous nucleotide substitutions at Gag 499 and nonsynonymous nucleotide substitutions at Gag 500 result in mutation in protease residue 12. We therefore observe a strong correlation between Gag 500 and PR 12, though this correlation holds little co-evolution information because both amino acid mutations are the manifestation of one set nucleotide substitutions. We do not observe strong Gag-PR correlations in the frameshift region (Gag 488–500 and PR 1–12), likely due to the observed conservation of PR residues 1–9 and 11. (DOC) [file pcbi.1004249.s012.doc]

**Table S4:** Top 1% of most strongly correlated pairs of Gag-PR positions

| **Gag Position** | **Pr Position** | **Gag Protein** | **Protease**  **PI-association** | **MI** | **P_xy_** | **P_xy_^0^** | **P_x_** | **P_y_** |
| --- | --- | --- | --- | --- | --- | --- | --- | --- |
| 431 | 82 | NC/p1 CS | Yes | 0.085 | 0.098 | 0.032 | 0.158 | 0.200 |
| 431 | 46 | NC/p1 CS | Yes | 0.071 | 0.085 | 0.027 | 0.158 | 0.170 |
| 431 | 10 | NC/p1 CS | Yes | 0.063 | 0.106 | 0.045 | 0.158 | 0.282 |
| 8 | 57 | MA | No | 0.058 | 0.023 | 0.002 | 0.024 | 0.086 |
| 264 | 76 | CA | Yes | 0.056 | 0.012 | 0.000 | 0.013 | 0.014 |
| 486 | 37 | p6 | No | 0.055 | 0.076 | 0.029 | 0.094 | 0.309 |
| 159 | 37 | CA | No | 0.053 | 0.163 | 0.093 | 0.300 | 0.309 |
| 443 | 35 | p1 | Yes | 0.048 | 0.030 | 0.006 | 0.030 | 0.204 |
| 465 | 76 | p6 | Yes | 0.046 | 0.012 | 0.000 | 0.020 | 0.014 |
| 375 | 37 | p2/NC CS | No | 0.046 | 0.163 | 0.097 | 0.313 | 0.309 |
| 119 | 37 | MA | No | 0.045 | 0.070 | 0.029 | 0.092 | 0.309 |
| 326 | 57 | CA | No | 0.044 | 0.038 | 0.008 | 0.088 | 0.086 |
| 65 | 43 | MA | Yes | 0.044 | 0.021 | 0.002 | 0.058 | 0.030 |
| 163 | 72 | CA | Yes | 0.044 | 0.037 | 0.008 | 0.052 | 0.157 |
| 453 | 36 | p1/p6 CS | Yes | 0.044 | 0.075 | 0.029 | 0.149 | 0.195 |
| 182 | 16 | CA | No | 0.044 | 0.018 | 0.001 | 0.050 | 0.023 |
| 486 | 24 | p6 | Yes | 0.043 | 0.022 | 0.003 | 0.094 | 0.027 |
| 456 | 24 | p6 | Yes | 0.043 | 0.027 | 0.005 | 0.194 | 0.027 |
| 410 | 20 | NC | Yes | 0.042 | 0.018 | 0.002 | 0.018 | 0.087 |
| 431 | 93 | NC/p1 CS | Yes | 0.042 | 0.104 | 0.053 | 0.158 | 0.334 |
| 67 | 93 | MA | Yes | 0.041 | 0.107 | 0.055 | 0.165 | 0.334 |
| 443 | 41 | p1 | No | 0.041 | 0.030 | 0.008 | 0.030 | 0.256 |
| 411 | 32 | NC | Yes | 0.041 | 0.023 | 0.003 | 0.117 | 0.028 |
| 443 | 77 | p1 | Yes | 0.040 | 0.030 | 0.008 | 0.030 | 0.261 |
| 118 | 66 | MA | Yes | 0.040 | 0.017 | 0.001 | 0.044 | 0.026 |
| 128 | 66 | MA/CA CS | Yes | 0.039 | 0.017 | 0.001 | 0.042 | 0.026 |
| 460 | 37 | p6 | No | 0.039 | 0.114 | 0.061 | 0.197 | 0.309 |
| 310 | 93 | CA | Yes | 0.039 | 0.110 | 0.059 | 0.176 | 0.334 |
| 348 | 35 | CA | Yes | 0.039 | 0.042 | 0.012 | 0.058 | 0.204 |
| 123 | 35 | MA | Yes | 0.039 | 0.038 | 0.010 | 0.050 | 0.204 |
| 46 | 41 | MA | No | 0.038 | 0.076 | 0.033 | 0.130 | 0.256 |
| 449 | 84 | p1/p6 CS | Yes | 0.038 | 0.026 | 0.004 | 0.076 | 0.050 |
| 403 | 63 | NC | Yes | 0.038 | 0.433 | 0.375 | 0.498 | 0.752 |
| 463 | 41 | p6 | No | 0.038 | 0.046 | 0.015 | 0.059 | 0.256 |
| ^†^500 | 12 | p6 | No | 0.038 | 0.020 | 0.002 | 0.025 | 0.088 |
