# Supplementary material for: Deep Sequencing of Protease Inhibitor Resistant HIV Patient Isolates Reveals Patterns of Correlated Mutations in Gag and Protease
Source: PLoS Comput Biol. 2015 Apr 20;11(4):e1004249. doi: 10.1371/journal.pcbi.1004249 (PMC4404092; doi:10.1371/journal.pcbi.1004249)
Supplement: S5 Table — (DOC) [file pcbi.1004249.s013.doc]

**Table S5:** Top 1% of most strongly correlated pairs of Gag-Gag positions

| **Gag Position 1** | **Gag Position 2** | **Gag Protein 1** | **Gag Protein 2** | **MI** | **P_xy_** | **P_xy_^0^** | **P_x_** | **P_y_** |
| --- | --- | --- | --- | --- | --- | --- | --- | --- |
| 228 | 248 | CA | CA | 0.214 | 0.160 | 0.048 | 0.168 | 0.288 |
| 159 | 280 | CA | CA | 0.144 | 0.219 | 0.102 | 0.300 | 0.341 |
| 46 | 75 | MA | MA | 0.108 | 0.069 | 0.012 | 0.130 | 0.094 |
| 123 | 443 | MA | p1 | 0.098 | 0.030 | 0.002 | 0.050 | 0.030 |
| 163 | 443 | CA | p1 | 0.097 | 0.030 | 0.002 | 0.052 | 0.030 |
| 12 | 46 | MA | MA | 0.095 | 0.121 | 0.052 | 0.398 | 0.130 |
| 348 | 443 | CA | p1 | 0.093 | 0.030 | 0.002 | 0.058 | 0.030 |
| 163 | 418 | CA | NC | 0.086 | 0.049 | 0.009 | 0.052 | 0.168 |
| 63 | 66 | MA | MA | 0.086 | 0.024 | 0.001 | 0.025 | 0.037 |
| 182 | 186 | CA | CA | 0.077 | 0.036 | 0.004 | 0.050 | 0.071 |
| 173 | 342 | CA | CA | 0.076 | 0.050 | 0.009 | 0.142 | 0.064 |
| 75 | 443 | MA | p1 | 0.074 | 0.030 | 0.003 | 0.094 | 0.030 |
| 119 | 443 | MA | p1 | 0.074 | 0.030 | 0.003 | 0.092 | 0.030 |
| 403 | 418 | NC | NC | 0.073 | 0.151 | 0.083 | 0.498 | 0.168 |
| 242 | 248 | CA | CA | 0.072 | 0.091 | 0.033 | 0.116 | 0.288 |
| 397 | 404 | NC | NC | 0.072 | 0.023 | 0.001 | 0.043 | 0.025 |
| 387 | 398 | NC | NC | 0.072 | 0.042 | 0.006 | 0.094 | 0.061 |
| 123 | 348 | MA | CA | 0.071 | 0.032 | 0.003 | 0.050 | 0.058 |
| 340 | 495 | CA | p6 | 0.067 | 0.111 | 0.048 | 0.317 | 0.151 |
| 79 | 81 | MA | MA | 0.066 | 0.075 | 0.025 | 0.255 | 0.097 |
| 390 | 401 | NC | NC | 0.066 | 0.051 | 0.010 | 0.118 | 0.083 |
| 173 | 248 | CA | CA | 0.065 | 0.101 | 0.041 | 0.142 | 0.288 |
| 123 | 163 | MA | CA | 0.064 | 0.030 | 0.003 | 0.050 | 0.052 |
| 130 | 268 | MA/CA CS | CA | 0.064 | 0.024 | 0.002 | 0.027 | 0.062 |
| 46 | 443 | MA | p1 | 0.064 | 0.030 | 0.004 | 0.130 | 0.030 |
| 46 | 119 | MA | MA | 0.063 | 0.054 | 0.012 | 0.130 | 0.092 |
| 146 | 148 | CA | CA | 0.063 | 0.041 | 0.008 | 0.190 | 0.043 |
| 165 | 256 | CA | CA | 0.063 | 0.020 | 0.001 | 0.030 | 0.026 |
| 28 | 122 | MA | MA | 0.062 | 0.128 | 0.061 | 0.304 | 0.199 |
| 8 | 326 | MA | CA | 0.060 | 0.024 | 0.002 | 0.024 | 0.088 |
| 82 | 84 | MA | MA | 0.060 | 0.174 | 0.098 | 0.293 | 0.336 |
| 163 | 348 | CA | CA | 0.059 | 0.030 | 0.003 | 0.052 | 0.058 |
| 218 | 219 | CA | CA | 0.057 | 0.028 | 0.004 | 0.029 | 0.130 |
| 248 | 460 | CA | p6 | 0.057 | 0.120 | 0.057 | 0.288 | 0.197 |
| 163 | 248 | CA | CA | 0.056 | 0.050 | 0.015 | 0.052 | 0.288 |
| 186 | 260 | CA | CA | 0.056 | 0.031 | 0.004 | 0.071 | 0.050 |
| 418 | 443 | NC | p1 | 0.055 | 0.030 | 0.005 | 0.168 | 0.030 |
| 111 | 443 | MA | p1 | 0.055 | 0.030 | 0.005 | 0.161 | 0.030 |
| 12 | 72 | MA | MA | 0.055 | 0.059 | 0.024 | 0.398 | 0.059 |
| 46 | 72 | MA | MA | 0.054 | 0.040 | 0.008 | 0.130 | 0.059 |
| 310 | 443 | CA | p1 | 0.053 | 0.030 | 0.005 | 0.176 | 0.030 |
| 53 | 332 | MA | CA | 0.053 | 0.015 | 0.001 | 0.030 | 0.015 |
| 373 | 378 | p2/NC CS | p2/NC CS | 0.052 | 0.105 | 0.051 | 0.383 | 0.133 |
| 182 | 223 | CA | CA | 0.051 | 0.048 | 0.016 | 0.050 | 0.315 |
| 58 | 443 | MA | p1 | 0.051 | 0.030 | 0.006 | 0.182 | 0.030 |
| 388 | 488 | NC | p6 | 0.050 | 0.026 | 0.003 | 0.099 | 0.031 |
| 163 | 374 | CA | p2/NC CS | 0.050 | 0.049 | 0.017 | 0.052 | 0.319 |
| 286 | 348 | CA | CA | 0.050 | 0.048 | 0.013 | 0.222 | 0.058 |
| 370 | 443 | CA/p2 CS | p1 | 0.050 | 0.030 | 0.006 | 0.190 | 0.030 |
| 443 | 460 | p1 | p6 | 0.050 | 0.030 | 0.006 | 0.030 | 0.197 |
| 219 | 248 | CA | CA | 0.050 | 0.088 | 0.037 | 0.130 | 0.288 |
| 186 | 303 | CA | CA | 0.049 | 0.020 | 0.002 | 0.071 | 0.022 |
| 427 | 468 | NC | p6 | 0.049 | 0.025 | 0.003 | 0.069 | 0.036 |
| 75 | 286 | MA | CA | 0.049 | 0.063 | 0.021 | 0.094 | 0.222 |
| 126 | 443 | MA | p6 | 0.048 | 0.030 | 0.006 | 0.196 | 0.030 |
| 441 | 443 | p1 | p1 | 0.048 | 0.030 | 0.006 | 0.210 | 0.030 |
| 126 | 242 | MA | CA | 0.048 | 0.067 | 0.023 | 0.196 | 0.116 |
| 443 | 470 | p1 | p6 | 0.048 | 0.030 | 0.006 | 0.030 | 0.203 |
| 148 | 173 | CA | CA | 0.048 | 0.034 | 0.006 | 0.043 | 0.142 |
| 191 | 418 | CA | NC | 0.048 | 0.032 | 0.006 | 0.036 | 0.168 |
| 18 | 332 | MA | CA | 0.048 | 0.015 | 0.001 | 0.041 | 0.015 |
| 264 | 465 | CA | p6 | 0.047 | 0.012 | 0.000 | 0.013 | 0.020 |
| 242 | 443 | CA | p1 | 0.047 | 0.026 | 0.004 | 0.116 | 0.030 |
| 460 | 499 | p6 | p6 | 0.047 | 0.034 | 0.007 | 0.197 | 0.037 |
| 163 | 460 | CA | p6 | 0.047 | 0.041 | 0.010 | 0.052 | 0.197 |
| 122 | 443 | MA | p1 | 0.047 | 0.030 | 0.006 | 0.199 | 0.030 |
| 375 | 486 | p2/NC CS | p6 | 0.047 | 0.073 | 0.030 | 0.313 | 0.094 |
| 191 | 428 | CA | NC/p1 CS | 0.046 | 0.019 | 0.001 | 0.036 | 0.036 |
| 75 | 348 | MA | CA | 0.046 | 0.033 | 0.006 | 0.094 | 0.058 |
| 147 | 268 | CA | CA | 0.046 | 0.050 | 0.016 | 0.255 | 0.062 |
| 312 | 425 | CA | NC | 0.046 | 0.071 | 0.029 | 0.314 | 0.092 |
