# Supplementary material for: Deep Sequencing of Protease Inhibitor Resistant HIV Patient Isolates Reveals Patterns of Correlated Mutations in Gag and Protease
Source: PLoS Comput Biol. 2015 Apr 20;11(4):e1004249. doi: 10.1371/journal.pcbi.1004249 (PMC4404092; doi:10.1371/journal.pcbi.1004249)
Supplement: S6 Table — Listed are the smallest atom-atom distances (only heavy atoms, excluding side chains) for the most strongly correlated pairs of residues from the three regions PR-PR, Gag-PR, Gag-Gag ranked by MI in representative structures. For structures with multiple chains, inter-chain distances were computed and the chain and atom combinations of the smallest calculated distance for each pair are listed. Pairs with atom-atom distances above 8Å are listed in gray. aMA: matrix monomer, PDB 2H3F; MA3: matrix trimer, PDB 1HIW; CA: capsid monomer, PDB 3MGE; CA2: capsid dimer, PDB 2M8L; CA5: capsid pentamer, PDB 3P05; CA6: capsid hexamer, PDB 3MGE; NC: nucleocapsid monomer, PDB 2EXF; PR2: protease dimer, PDB 1ODW. bFor PDB files with multiple structural models, the model number with the smallest atom-atom distance is listed. (DOC) [file pcbi.1004249.s014.doc]

**Table S6:** Smallest all-atom distances between strongly correlated pairs of residues in Gag and protease as identified by mutual information

| **Protein** | **Pos 1** | **Pos 2** | **Res 1** | **Res 2** | **MI** | **Smallest Rij (Å)** | **Structure^a^** | **PDB Model^b^** | **Chains** | **Atoms** |
| --- | --- | --- | --- | --- | --- | --- | --- | --- | --- | --- |
| CA | 228 | 248 | MET | GLY | 0.21 | **3.1** | CA2 | 22 | BB | CE-O |
| CA | 159 | 280 | VAL | THR | 0.14 | **6.4** | CA2 | 39 | AA | O-OG1 |
| PR | 30 | 88 | ASP | ASN | 0.13 | **3.7** | PR2 | 0 | AA | CA-OD1 |
| MA | 46 | 75 | VAL | LEU | 0.11 | **6.7** | MA3 | 0 | AC | CA-CD2 |
| MA | 12 | 46 | GLU | VAL | 0.10 | **16.6** | MA | 11 | AA | OE1-CG1 |
| PR | 54 | 82 | ILE | VAL | 0.09 | **8.2** | PR2 | 0 | BB | CD1-O |
| MA | 63 | 66 | GLN | PRO | 0.09 | **3.0** | MA | 14 | AA | O-CG |
| CA | 182 | 186 | GLN | THR | 0.08 | **2.4** | CA2 | 10 | AA | OE1-OG1 |
| CA | 173 | 342 | SER | THR | 0.08 | **14.3** | CA2 | 69 | AB | OG-O |
| NC | 403 | 418 | GLY | ASN | 0.07 | **5.5** | NC | 9 | AA | CA-ND2 |
| CA | 242 | 248 | THR | GLY | 0.07 | **7.6** | CA5 | 0 | EE | O-CA |
| NC | 397 | 404 | LYS | LYS | 0.07 | **6.6** | NC | 5 | AA | O-O |
| NC | 387 | 398 | THR | GLY | 0.07 | **13.8** | NC | 9 | AA | O-O |
| PR | 73 | 90 | GLY | LEU | 0.07 | **10.6** | PR2 | 0 | BB | CA-CA |
| PR | 46 | 82 | MET | VAL | 0.07 | **14.6** | PR2 | 0 | BA | O-CG2 |
| MA | 79 | 81 | TYR | THR | 0.07 | **4.0** | MA | 19 | AA | O-CA |
| NC | 390 | 401 | ASN | LYS | 0.07 | **16.1** | NC | 5 | AA | ND2-NZ |
| CA | 173 | 248 | SER | GLY | 0.07 | **20.4** | CA5 | 0 | BB | O-O |
| MA | 46 | 119 | VAL | ALA | 0.06 | **17.7** | MA | 14 | AA | CG2-CB |
| CA | 146 | 148 | ALA | SER | 0.06 | **3.9** | CA5 | 0 | DD | O-CA |
| CA | 165 | 256 | SER | ILE | 0.06 | **18.0** | CA2 | 11 | AB | OG-CD1 |
| MA | 28 | 122 | GLN | THR | 0.06 | **34.6** | MA | 6 | AA | O-CG2 |
| PR | 24 | 74 | LEU | THR | 0.06 | **12.6** | PR2 | 0 | BB | CD2-O |
| MA | 82 | 84 | ILE | VAL | 0.06 | **4.1** | MA | 16 | AA | O-CA |
| CA | 163 | 348 | ALA | THR | 0.06 | **16.7** | CA5 | 0 | AE | CB-OG1 |
| CA | 218 | 219 | VAL | HIS | 0.06 | **2.7** | CA | 0 | AA | O-CA |
| CA | 163 | 248 | ALA | GLY | 0.06 | **30.9** | CA2 | 11 | AB | O-O |
| CA | 186 | 260 | THR | GLU | 0.06 | **12.6** | CA5 | 0 | AE | OG1-OE1 |
| MA | 12 | 72 | GLU | SER | 0.06 | **21.0** | MA3 | 0 | CB | OE1-CA |
| MA | 46 | 72 | VAL | SER | 0.05 | **3.6** | MA3 | 0 | BA | O-CB |
| PR | 35 | 36 | GLU | MET | 0.05 | **2.7** | PR2 | 0 | AA | O-CA |
| CA | 182 | 223 | GLN | ILE | 0.05 | **18.8** | CA | 0 | AA | OE1-CD1 |
| CA | 286 | 348 | ARG | THR | 0.05 | **18.2** | CA5 | 0 | DD | O-O |
| CA | 219 | 248 | HIS | GLY | 0.05 | **12.5** | CA2 | 95 | BA | NE2-O |
| CA | 186 | 303 | THR | THR | 0.05 | **16.5** | CA2 | 78 | AA | O-CA |
| PR | 69 | 84 | HIS | ILE | 0.05 | **13.1** | PR2 | 0 | BB | O-O |
| CA | 148 | 173 | SER | SER | 0.05 | **9.8** | CA5 | 0 | BA | O-O |
| CA | 147 | 268 | ILE | LEU | 0.05 | **18.5** | CA2 | 60 | AA | CD1-CA |
| PR | 24 | 46 | LEU | MET | 0.05 | **20.1** | PR2 | 0 | AB | O-O |
| PR | 24 | 82 | LEU | VAL | 0.04 | **8.6** | PR2 | 0 | BB | CA-CG2 |
| PR | 13 | 33 | ILE | LEU | 0.04 | **4.3** | PR2 | 0 | AA | CD1-CD2 |
| PR | 10 | 93 | LEU | ILE | 0.04 | **11.0** | PR2 | 0 | AA | O-CD1 |
| PR | 12 | 19 | THR | LEU | 0.04 | **3.9** | PR2 | 0 | BB | CG2-CB |
