# Supplementary material for: Deep Sequencing of Protease Inhibitor Resistant HIV Patient Isolates Reveals Patterns of Correlated Mutations in Gag and Protease
Source: PLoS Comput Biol. 2015 Apr 20;11(4):e1004249. doi: 10.1371/journal.pcbi.1004249 (PMC4404092; doi:10.1371/journal.pcbi.1004249)
Supplement: S7 Table — Some of the distance calculations used in Tables 5 and S6 are derived from ensembles of structures. Shown here are the minimum, mean, and maximum atom-atom distances for all affected position pairs calculated from each PDB with an ensemble of structural models. For distances <8Å, we find there to be little variability across structural models. (DOC) [file pcbi.1004249.s015.doc]

**Table S7:** Distribution of atom-atom distances among models in PDBs derived from NMR

| **PDB** | **Structure** | **Gag Pos 1** | **Gag Pos 2** | **PDB**  **Pos 1** | **PDB**  **Pos 2** | **Res 1** | **Res 2** | **MI** | **Min**  **Rij (Å)** | **Avg**  **Rij (Å)** | **Max**  **Rij (Å)** |
| --- | --- | --- | --- | --- | --- | --- | --- | --- | --- | --- | --- |
| 2M8L | CA2 | 146 | 148 | 14 | 16 | ALA | SER | 0.06 | 4.3 | 4.3 | 4.3 |
| 2M8L | CA2 | 147 | 268 | 15 | 136 | ILE | LEU | 0.05 | 18.5 | 18.8 | 19.5 |
| 2M8L | CA2 | 148 | 173 | 16 | 41 | SER | SER | 0.05 | 12.9 | 13.2 | 13.4 |
| 2M8L | CA2 | 159 | 280 | 27 | 148 | VAL | THR | 0.14 | 6.4 | 14.3 | 22.9 |
| 2M8L | CA2 | 163 | 248 | 31 | 116 | ALA | GLY | 0.06 | 30.9 | 33.3 | 33.3 |
| 2M8L | CA2 | 163 | 348 | 31 | 216 | ALA | THR | 0.06 | 21.6 | 28.1 | 35.6 |
| 2M8L | CA2 | 165 | 256 | 33 | 124 | SER | ILE | 0.06 | 18.0 | 27.0 | 27.4 |
| 2M8L | CA2 | 173 | 248 | 41 | 116 | SER | GLY | 0.07 | 20.7 | 20.7 | 20.7 |
| 2M8L | CA2 | 173 | 342 | 41 | 210 | SER | THR | 0.08 | 14.3 | 27.0 | 41.4 |
| 2M8L | CA2 | 182 | 186 | 50 | 54 | GLN | THR | 0.08 | 2.4 | 2.7 | 3.0 |
| 2M8L | CA2 | 182 | 223 | 50 | 91 | GLN | ILE | 0.05 | 20.7 | 21.7 | 22.4 |
| 2M8L | CA2 | 186 | 260 | 54 | 128 | THR | GLU | 0.06 | 13.9 | 15.0 | 15.0 |
| 2M8L | CA2 | 186 | 303 | 54 | 171 | THR | THR | 0.05 | 16.5 | 28.8 | 35.6 |
| 2M8L | CA2 | 218 | 219 | 86 | 87 | VAL | HIS | 0.06 | 2.8 | 2.8 | 2.8 |
| 2M8L | CA2 | 219 | 248 | 87 | 116 | HIS | GLY | 0.05 | 12.5 | 16.3 | 17.1 |
| 2M8L | CA2 | 228 | 248 | 96 | 116 | MET | GLY | 0.21 | 3.1 | 4.6 | 5.8 |
| 2M8L | CA2 | 242 | 248 | 110 | 116 | THR | GLY | 0.07 | 7.7 | 7.7 | 7.7 |
| 2M8L | CA2 | 286 | 348 | 154 | 216 | ARG | THR | 0.05 | 18.2 | 18.2 | 18.2 |
| 2H3F | MA | 12 | 46 | 12 | 46 | GLU | VAL | 0.10 | 16.6 | 17.2 | 17.8 |
| 2H3F | MA | 12 | 72 | 12 | 72 | GLU | SER | 0.06 | 24.9 | 25.8 | 26.8 |
| 2H3F | MA | 28 | 122 | 28 | 122 | GLN | THR | 0.06 | 34.6 | 42.6 | 49.4 |
| 2H3F | MA | 46 | 72 | 46 | 72 | VAL | SER | 0.05 | 13.9 | 15.2 | 16.7 |
| 2H3F | MA | 46 | 75 | 46 | 75 | VAL | LEU | 0.11 | 10.0 | 10.9 | 12.6 |
| 2H3F | MA | 46 | 119 | 46 | 119 | VAL | ALA | 0.06 | 17.7 | 26.4 | 36.6 |
| 2H3F | MA | 63 | 66 | 63 | 66 | GLN | PRO | 0.09 | 3.0 | 3.2 | 4.4 |
| 2H3F | MA | 79 | 81 | 79 | 81 | TYR | THR | 0.07 | 4.0 | 4.2 | 4.3 |
| 2H3F | MA | 82 | 84 | 82 | 84 | ILE | VAL | 0.06 | 4.1 | 4.1 | 4.3 |
| 2EXF | NC | 387 | 398 | 24 | 35 | THR | GLY | 0.07 | 13.8 | 14.1 | 14.7 |
| 2EXF | NC | 390 | 401 | 27 | 38 | ASN | LYS | 0.07 | 16.1 | 18.1 | 19.0 |
| 2EXF | NC | 397 | 404 | 34 | 41 | LYS | LYS | 0.07 | 6.6 | 6.8 | 7.0 |
| 2EXF | NC | 403 | 418 | 40 | 55 | GLY | ASN | 0.07 | 5.5 | 11.3 | 13.6 |
