# Supplementary material for: Deep Sequencing of Protease Inhibitor Resistant HIV Patient Isolates Reveals Patterns of Correlated Mutations in Gag and Protease
Source: PLoS Comput Biol. 2015 Apr 20;11(4):e1004249. doi: 10.1371/journal.pcbi.1004249 (PMC4404092; doi:10.1371/journal.pcbi.1004249)
Supplement: S8 Table — We show a comparison between PR-PR pair rankings calculated using Fisher’s exact test on a MSA provided by the Stanford HIVDB [24] dated 4/29/2013 and the permutation test presented in [3]. (DOC) [file pcbi.1004249.s016.doc]

**Table S8:** PR-PR pairs ranked by Fisher exact test p-value calculated from 2013 HIVDB sequences

| **Pair** | **Shafer Rank** | **Fisher Rank** | **Fisher P-value** |
| --- | --- | --- | --- |
| 54V-82A | 1 | 1 | 5.6E-298 |
| 30N-88D | 2 | 2 | 5.0E-249 |
| 35D-36I | 3 | 9 | 5.2E-128 |
| 71V-90M | 4 | 4 | 5.1E-176 |
| 10I-54V | 5 | 8 | 2.6E-131 |
| 10I-90M | 6 | 17 | 5.2E-97 |
| 10I-82A | 7 | 6 | 1.8E-137 |
| 71V-82A | 8 | 10 | 1.5E-118 |
| 54V-71V | 9 | 7 | 1.6E-136 |
| 10I-71V | 10 | 15 | 1.8E-100 |
| 20R-36I | 11 | 3 | 9.9E-205 |
| 77I-93L | 12 | 23 | 2.9E-74 |
| 46L-82A | 13 | 18 | 1.0E-105 |
| 73S-90M | 14 | 5 | 1.7E-150 |
| 36I-62V | 15 | 34 | 1.7E-56 |
| 84V-90M | 16 | 14 | 5.2E-101 |
| 46I-90M | 17 | 18 | 4.1E-88 |
| 10I-84V | 18 | 28 | 3.6E-63 |
| 62V-90M | 19 | 30 | 1.8E-60 |
| 71V-73S | 20 | 19 | 7.5E-84 |
| 20R-54V | 21 | 27 | 1.4E-63 |
| 63P-90M | 22 | 16 | 1.4E-99 |
| 54V-90M | 23 | 26 | 2.1E-64 |
| 46L-54V | 24 | 25 | 2.2E-69 |
| 90M-93L | 25 | 28 | 2.6E-51 |
| 24I-54V | 26 | 20 | 8.7E-84 |
| 36I-54V | 27 | 45 | 8.5E-48 |
| 24I-82A | 28 | 21 | 4.9E-79 |
| 10I-46I | 29 | 50 | 5.8E-43 |
| 20R-35D | 30 | 41 | 4.4E-50 |
| 46I-84V | 31 | 29 | 3.7E-63 |
| 71V-84V | 32 | 37 | 1.3E-53 |
| 20R-82A | 33 | 43 | 3.7E-48 |
| 10I-93L | 34 | 62 | 2.4E-37 |
| 10I-62V | 35 | 51 | 8.9E-43 |
| 35D-37D | 36 | 97 | 1.1E-28 |
| 48V-82A | 37 | 22 | 4.7E-75 |
| 20I-90M | 38 | 48 | 9.7E-45 |
| 24I-46L | 39 | 46 | 2.0E-47 |
| 62V-93L | 40 | 74 | 8.6E-35 |
